# Supplementary figures and images for: Integrated multi-modal data analysis for computational modeling of healthy and location-dependent myocardial infarction conditions in porcine hearts
Source: PLoS Comput Biol. 2026 Mar 16;22(3):e1013688. doi: 10.1371/journal.pcbi.1013688 (PMC13008256; doi:10.1371/journal.pcbi.1013688)

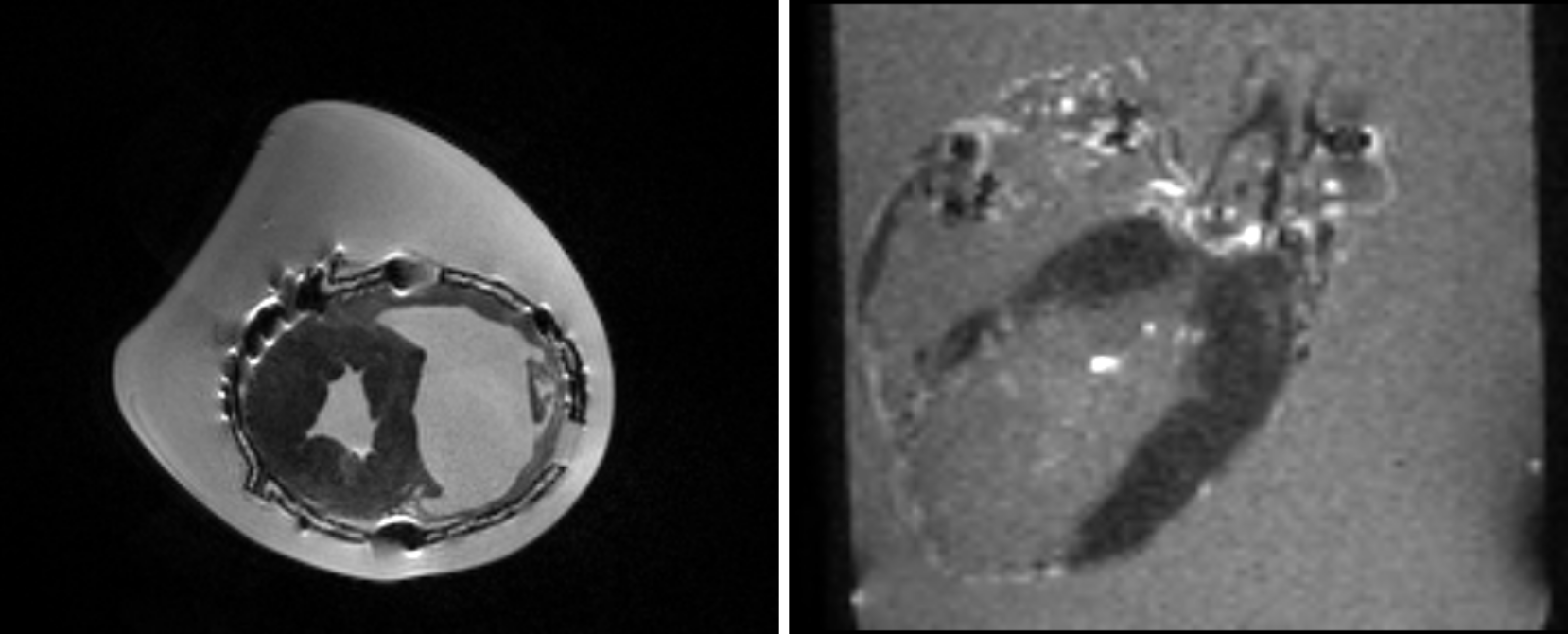

Supplement: S1 Fig — Representative transverse view of the healthy porcine heart (pig 3) acquired at KUL (left) and coronal view of the LAD-occlusion MI model (pig 14) acquired at SERMAS (right). DW-CMR: Diffusion-weighted cardiac magnetic resonance, KUL: Katholieke Universiteit Leuven, SERMAS: Servicio Madrileño de Salud, LAD: Left anterior descending, MI: Myocardial infarction. (TIFF) [file pcbi.1013688.s001.tiff]

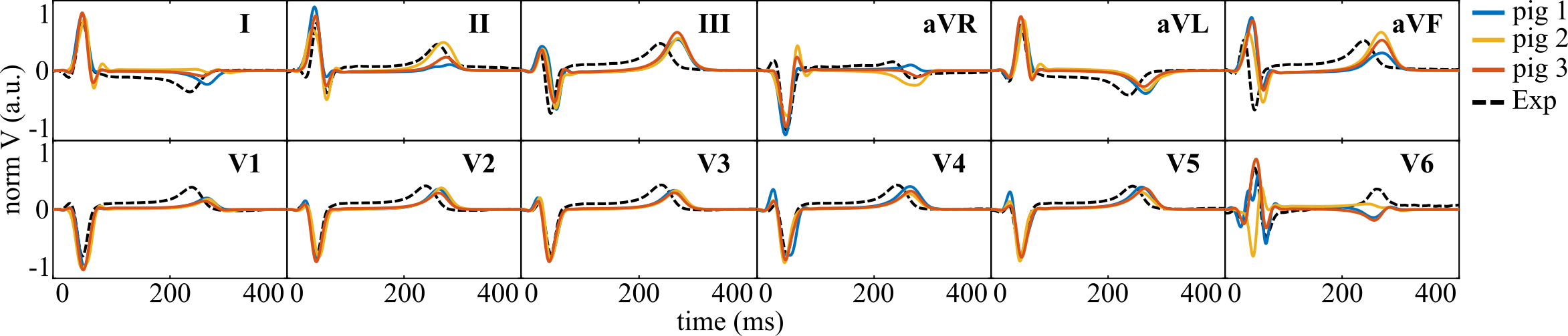

Supplement: S2 Fig — The complete simulated ECGs obtained for the three healthy pigs 1–3 when using the adjusted CS, APDSH, and s-RBM are shown together with the experimental ECG of pig 15. ECG: Electrocardiogram, CS: Conduction system, APDSH: Action potential duration spatial heterogeneities, s-RBM: Standard rule-based model. (TIFF) [file pcbi.1013688.s002.tiff]

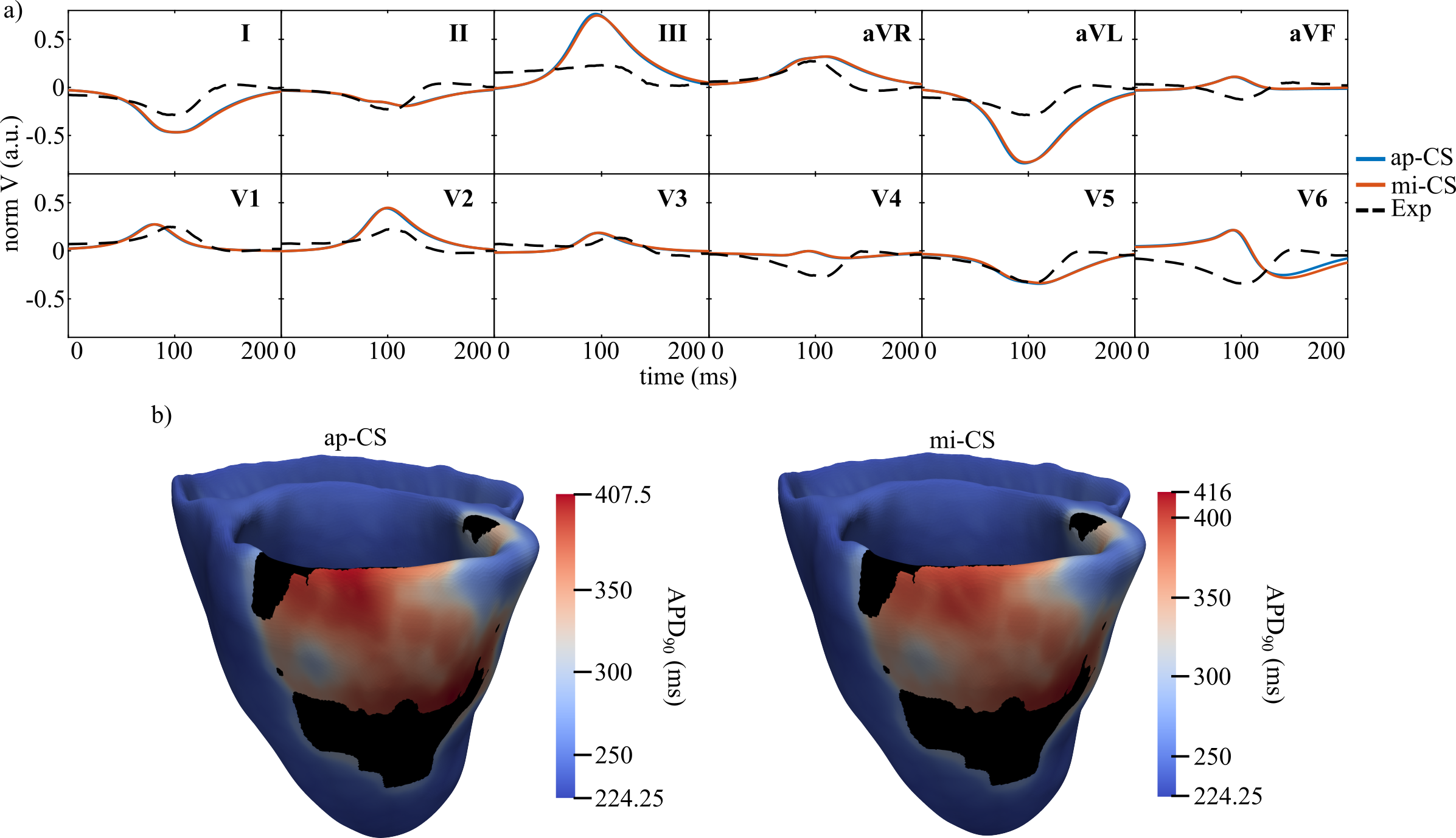

Supplement: S3 Fig — a- Experimental and simulated T waves for pig 6 when ap-CS and mi-CS were used. b- Simulated APD90 maps for pig 6 when the activation was generated by ap-CS and mi-CS. MI: Myocardial infarction, ap-CS: Intramyocardial, porcine conduction system with Purkinje in the septal wall of the right ventricle and the end of the left bundle branches closer to the apex of the left ventricle, mi-CS: ap-CS with altered spatial distribution of Purkinje fibers following infarction, APD90: Action potential duration at 90% repolarization. (TIFF) [file pcbi.1013688.s003.tiff]

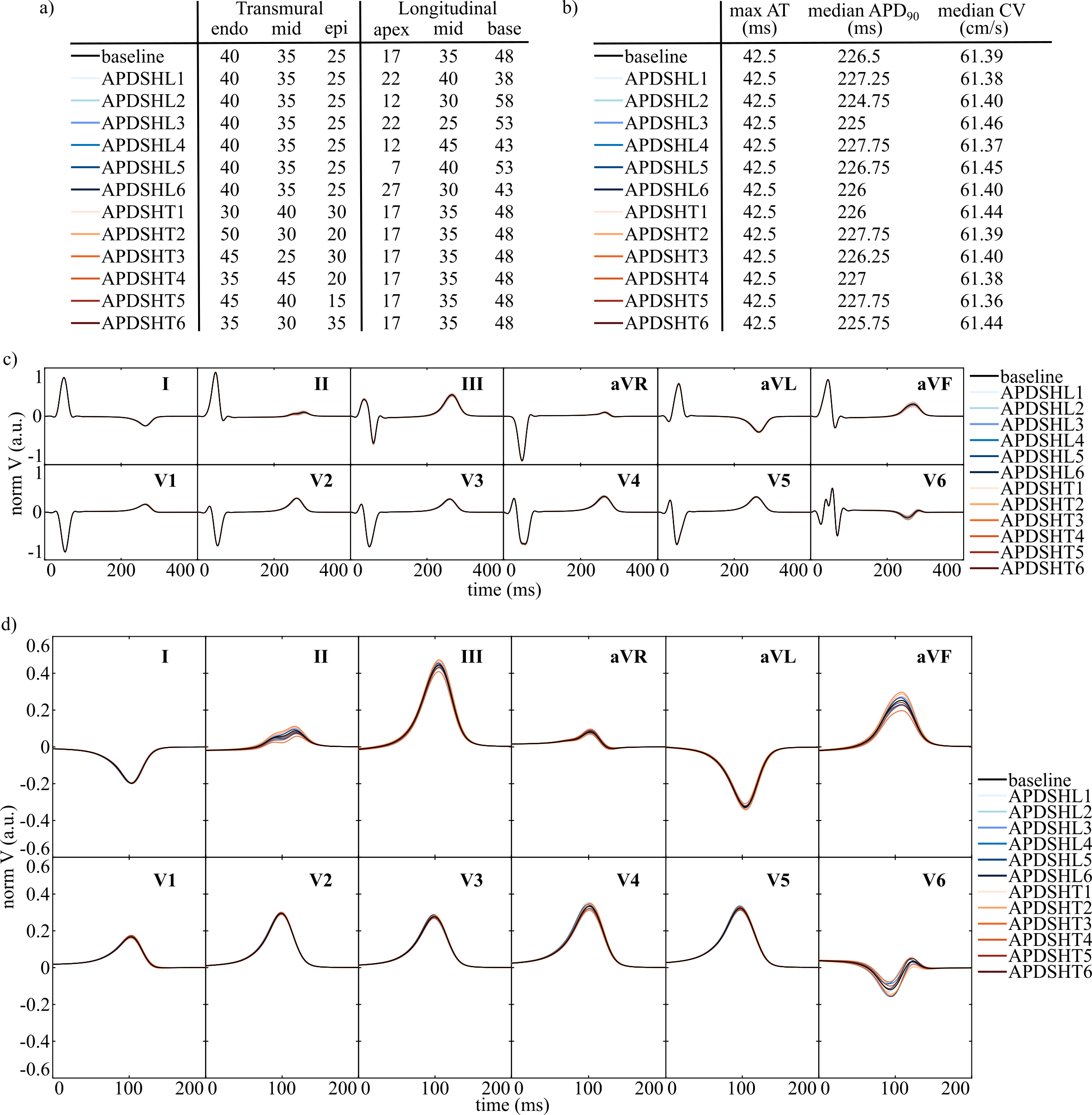

Supplement: S4 Fig — a- Twelve APDSH configurations were evaluated against the baseline APDSH, which was defined using transmural and longitudinal ratios adapted from non-porcine sources. Specifically, APDSH variations were generated by decreasing the size of one layer by 10% while simultaneously increasing the remaining two layers by 5% each; this was performed independently for both transmural and longitudinal directions. b- Maximum AT and median APD90 and CV values obtained at the anterior view of models with different APDSH configurations when simulated with a 1000 ms cycle length. c- Simulated ECGs demonstrating no variation in QRS complexes and negligible alterations in T waves across all tested APDSH ratios. d- Magnified simulated T-waves highlighting the preservation of T-wave morphology, with only minimal amplitude fluctuations primarily observed in leads II, III, aVF, and V4–V6. APDSH: Action potential duration spatial heterogeneities, ECG: Electrocardiogram, AT: Activation time, APD90: Action potential duration at 90% repolarization, CV: Conduction velocity. (TIFF) [file pcbi.1013688.s004.tiff]

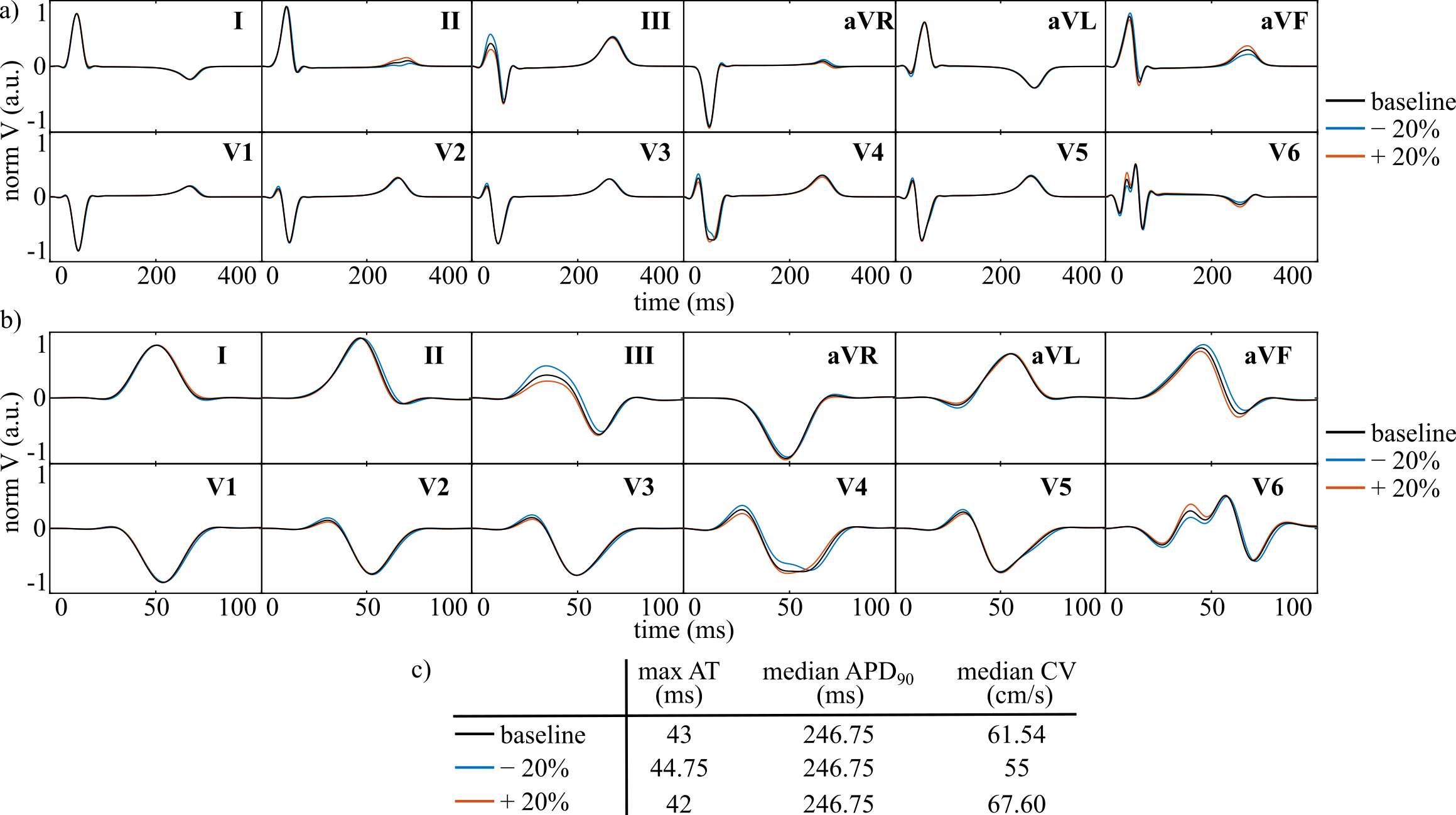

Supplement: S5 Fig — The baseline LDC of the healthy myocardium was varied by ±20% in pig 1. a- Complete simulated ECG signals. b- Magnified simulated QRS complexes. c- Simulated maximum AT and median APD90 and CV values obtained at the anterior face of the BiV model of pig 1. Simulated ECG and electrophysiological outputs were obtained under in silico pacing with 769 ms and 1000 ms cycle length, respectively. LDC: Longitudinal diffusion coefficient, ECG: Electrocardiogram, AT: Activation time, APD90: Action potential duration at 90% repolarization, CV: Conduction velocity, BiV: Biventricular. (TIFF) [file pcbi.1013688.s005.tiff]

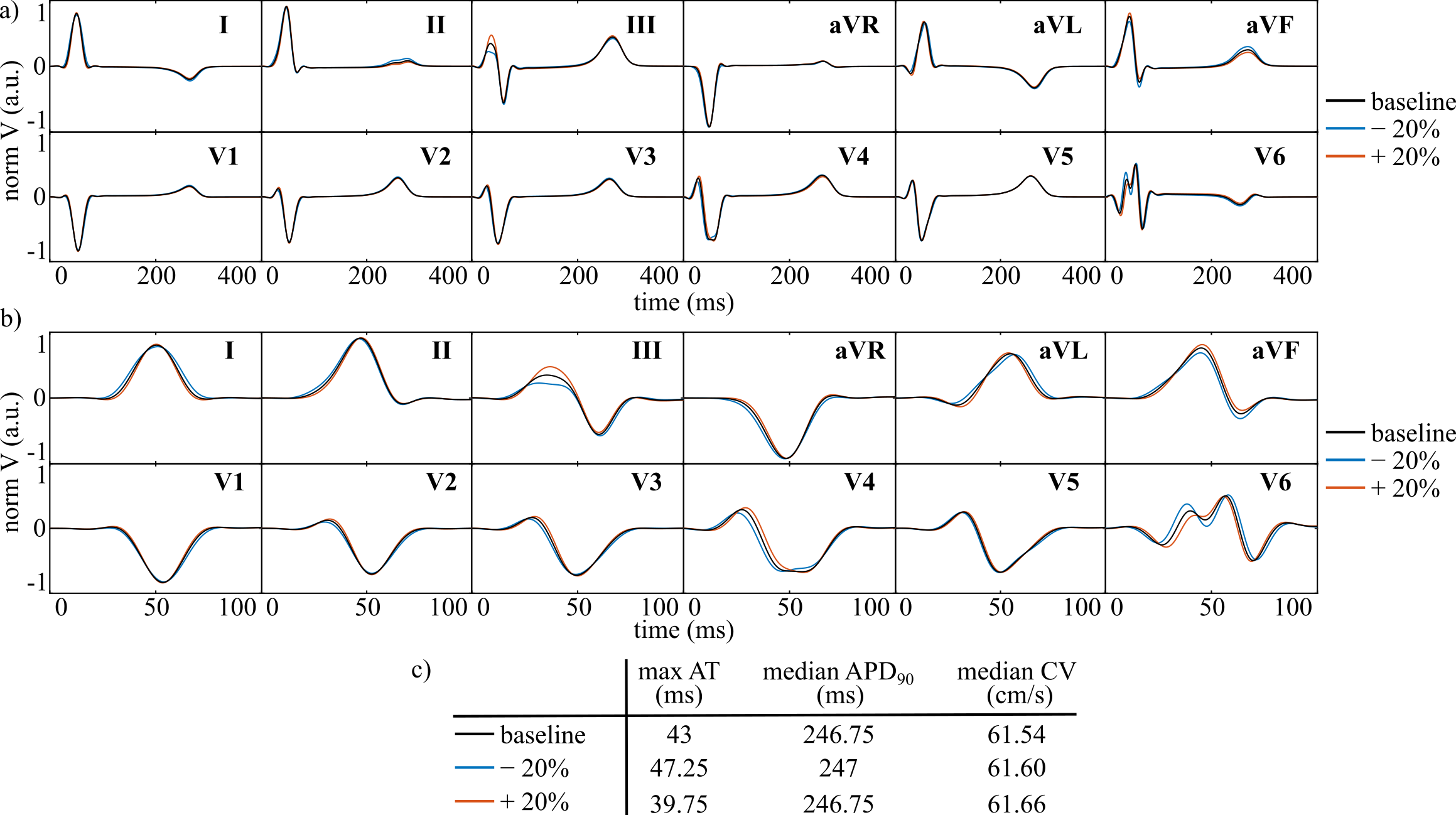

Supplement: S6 Fig — The baseline LDC of the CS was varied by ±20% in pig 1. a- Complete simulated ECG signals. b- Magnified simulated QRS complexes. c- Simulated maximum AT and median APD90 and CV values obtained at the anterior face of the BiV model of pig 1. Simulated ECG and electrophysiological outputs were obtained under in silico pacing with 769 ms and 1000 ms cycle length, respectively. CS: Conduction system, LDC: Longitudinal diffusion coefficient, ECG: Electrocardiogram, AT: Activation time, APD90: Action potential duration at 90% repolarization, CV: Conduction velocity, BiV: Biventricular. (TIFF) [file pcbi.1013688.s006.tiff]

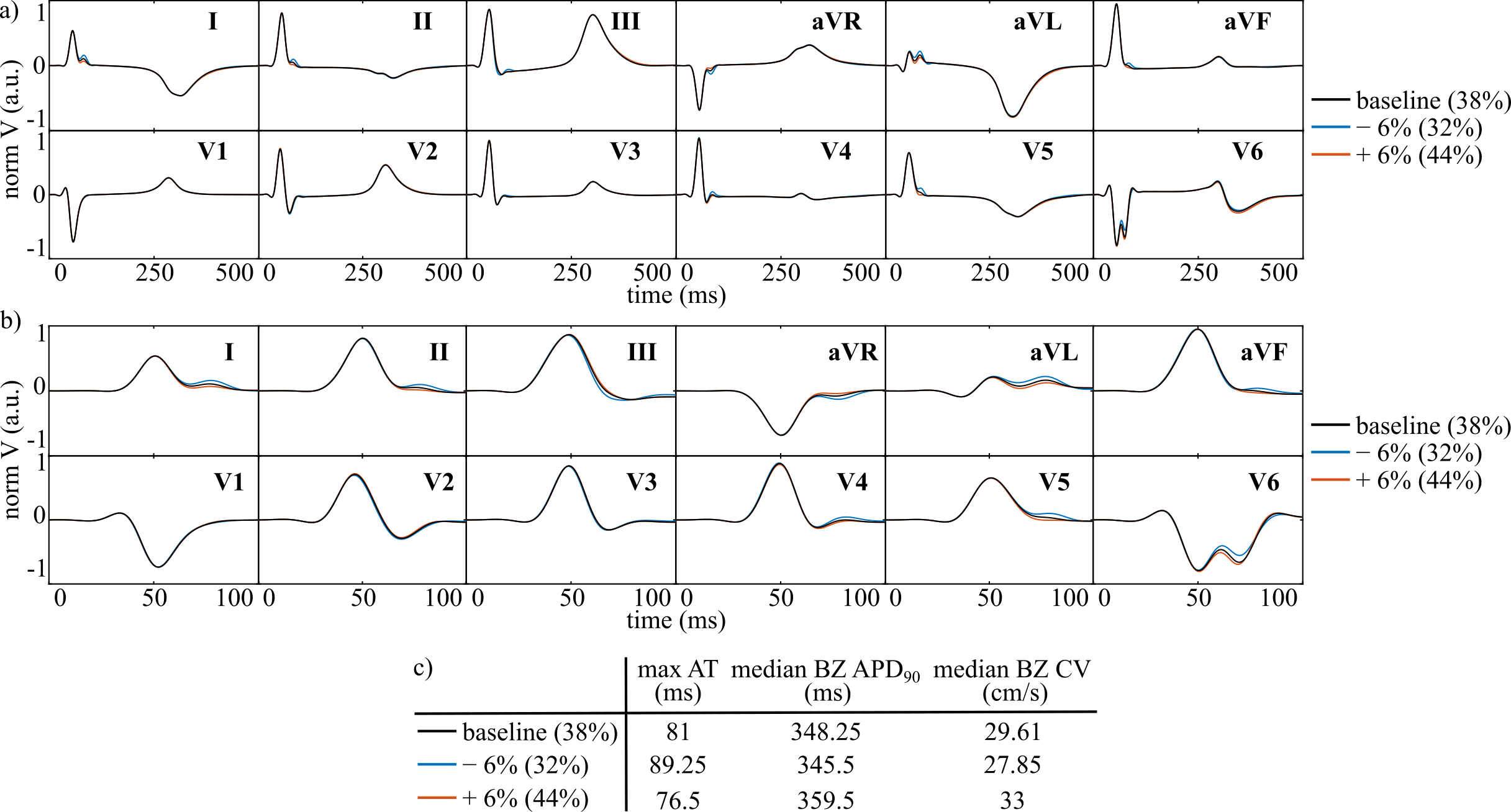

Supplement: S7 Fig — The gNa value applied to the BZ (38% of the healthy, default value) was varied by ±6% (32% and 44% of the healthy, default value) in pig 6. a- Complete simulated ECG signals. b- Magnified simulated QRS complexes. c- Simulated maximum ventricular AT and median APD90 and CV values within the BZ. Simulated ECG and electrophysiological outputs were both obtained under in silico pacing with 1000 ms cycle length. BZ: Border zone, INa: Sodium current, gNa: Maximum conductance of the INa, ECG: Electrocardiogram, AT: Activation time, APD90: Action potential duration at 90% repolarization, CV: Conduction velocity. (TIFF) [file pcbi.1013688.s007.tiff]
