# Supplementary material for: Integrated multi-modal data analysis for computational modeling of healthy and location-dependent myocardial infarction conditions in porcine hearts
Source: PLoS Comput Biol. 2026 Mar 16;22(3):e1013688. doi: 10.1371/journal.pcbi.1013688 (PMC13008256; doi:10.1371/journal.pcbi.1013688)
Supplement: Attachment — Submitted filename: Response_to_Reviewers.pdf [file pcbi.1013688.s008.pdf]

February 3, 2026

Re: Re-submission of manuscript "Integrated multi-modal data analysis for computational modeling of healthy and location-dependent myocardial infarction conditions in porcine hearts", with reference PCOMPBIOL-D-25-02241

Ricardo M. Rosales
University of Zaragoza
Zaragoza, Spain
rrosales@unizar.es

Editor in Chief
PLoS Computational Biology

Dear Editor,

We would like to thank you for the opportunity to revise our manuscript, "Integrated multi-modal data analysis for computational modeling of healthy and location-dependent myocardial infarction conditions in porcine hearts". We appreciate the careful review and the constructive suggestions provided by the Reviewers. We believe the manuscript has been substantially strengthened by incorporating these modifications.

The primary revisions are summarized below:

- Enhanced clarity and accessibility: We have incorporated several supporting figures and ensured that all abbreviations are defined within each figure and table caption. Furthermore, we have refined the tables and expanded the text to better illustrate the integration of experimental data into our modeling pipeline while clarifying potential use cases and the inherent limitations of the developed models.
- Robustness and uncertainty analysis: To strengthen the validation of our models, we have explicitly detailed the rationale behind our parameter selection. Additionally, we have performed a sensitivity analysis to address concerns regarding modeling robustness, particularly where cross-species data were used.
- Structural refinements: The manuscript has been restructured and edited for improved logical flow and clarity, with a specific focus on the Discussion section.
- Expanded context and literature review: Both the Introduction and Discussion sections have been extended with recent citations to provide a more thorough overview of the current state of the art in preclinical computational cardiac modeling.
- Journal compliance and data transparency: We have ensured full adherence to the journal's formatting guidelines. Notably, we added a new supporting figure (S3 Figure) to eliminate a previous "data not shown" statement, converted all figures to high-resolution .tiff format, resolved potential copyright requirements, and included a properly formatted funding statement.

Following this letter, we provide a point-by-point response (in blue) to the Reviewers' comments, indicating the specific changes made. For convenience, we have included the new images and references directly within the response to facilitate the review process.

This revision has been prepared in consultation with all co-authors, each of whom has approved the final version. We thank the Editor and the Reviewers once again for their valuable feedback.

Yours sincerely,
Ricardo M. Rosales, on behalf of all authors

Revision of manuscript PCOMPBIOL-D-25-02241

Journal Requirements:

1) We ask that a manuscript source file is provided at Revision. Please upload your manuscript file as a .doc, .docx, .rtf or .tex. If you are providing a .tex file, please upload it under the item type u2018LaTeX Source Fileu2019 and leave your .pdf version as the item type u2018Manuscriptu2019.

The documents have been provided as indicated.

2) We noticed that you used the phrase 'data not shown' in the manuscript. We do not allow these references, as the PLOS data access policy requires that all data be either published with the manuscript or made available in a publicly accessible database. Please amend the supplementary material to include the referenced data or remove the references.

We have added a supporting S3 Figure with the referenced data.

3) Please upload all main figures as separate Figure files in .tif or .eps format. For more information about how to convert and format your figure files please see our guidelines:

We converted all Figures to .tiff format.

4) Some material included in your submission may be copyrighted. According to PLOSu2019s copyright policy, authors who use figures or other material (e.g., graphics, clipart, maps) from another author or copyright holder must demonstrate or obtain permission to publish this material under the Creative Commons Attribution 4.0 International (CC BY 4.0) License used by PLOS journals. Please closely review the details of PLOSu2019s copyright requirements here: PLOS Licenses and Copyright. If you need to request permissions from a copyright holder, you may use PLOS's Copyright Content Permission form. Please respond directly to this email and provide any known details concerning your material's license terms and permissions required for reuse, even if you have not yet obtained copyright permissions or are unsure of your material's copyright compatibility. Once you have responded and addressed all other outstanding technical requirements, you may resubmit your manuscript within Editorial Manager. Potential Copyright Issues:
- Please confirm (a) that you are the photographer of Figure 1, or (b) provide written permission from the photographer to publish the photo(s) under our CC BY 4.0 license.

We have attached PLOS's Copyright Content Permission form signed by the copyright holder of the photographs in Figure 1.

- Figure 1. Please confirm whether you drew the images / clip-art within the figure panels by hand. If you did not draw the images, please provide (a) a link to the source of the images or icons and their license / terms of use; or (b) written permission from the copyright holder to publish the images or icons under our CC BY 4.0 license. Alternatively, you may replace the images with open source alternatives. See these open source resources you may use to replace images / clip-art: - https://commons.wikimedia.org - https://openclipart.org/.

Figure 1 was updated. Now it contains a combination of original hand-drawn elements and open-source icons. We have updated the figure to ensure all external elements are from public domain sources. Below are the specific sources and licensing details:

- MRI machine: Obtained from `https://openclipart.org/detail/284918/mri`.
- Heart: Obtained from `https://openclipart.org/detail/291180/realistic-red-heart`.
- Pig: Obtained from `https://openclipart.org/detail/314293/pig-silhouette`.
- Vaccine: Obtained from `https://openclipart.org/detail/330397/joel-lundgren`.

- Camera: Obtained from `https://commons.wikimedia.org/wiki/File:Video_camera_icon.svg`.
- Emitting light: Replaced with `https://openclipart.org/detail/185271/light-bulb-icon-2`.
- Monitor: Replaced with `https://openclipart.org/detail/35353/tango-utilities-system-monitor`.
- Heart photographs: These were provided by our co-authors (panel c and f). We have attached the signed PLOS "Copyright Content Permission" form to the submission, granting permission to publish these images under the CC BY 4.0 license.

All other elements within Figure 1 were generated from our own data or drawn by hand.

5) Figures 2, 3, 4, 5, 8, 9, 11, and Supporting Figure 3. Please confirm whether you drew the images / clip-art within the figure panels by hand. If you did not draw the images, please provide (a) a link to the source of the images or icons and their license / terms of use; or (b) written permission from the copyright holder to publish the images or icons under our CC BY 4.0 license. Alternatively, you may replace the images with open source alternatives. See these open source resources you may use to replace images / clip-art: - https://commons.wikimedia.org - https://openclipart.org/.

We confirm that all figures mentioned are original products of our research. No third-party clip art or external content was used in these panels; We created these figures based exclusively on our experimental data and results.

6) Please ensure that the funders and grant numbers match between the Financial Disclosure field and the Funding Information tab in your submission form. Note that the funders must be provided in the same order in both places as well. State the initials, alongside each funding source, of each author to receive each grant. For example: "This work was supported by the National Institutes of Health (NUMBER to AM; NUMBER to CJ) and the National Science Foundation (NUMBER to AM).". If you did not receive any funding for this study, please simply state: u201cThe authors received no specific funding for this work.u201d

In accordance with the requested guidelines, a funding statement has been incorporated into both the manuscript and the "Funding Information" section of the submission portal.

Response to Reviewer's comments

Reviewer 1:

Rosales et al. build sophisticated computer models of pig cardiac electrophysiology based on rich experimental data sets including myocardial infarction. They then study the sensitivity of selected simulation results (activation times, conduction velocity, ECG) on specific model parameters. Some of them are tuned then and a few comparisons to measured data are shown for validation purposes. The manuscript is very extensive; in general well written and supported by helpful figures an tables.

We are grateful to the Reviewer for the revisions and for the helpful and constructive comments, which we addressed as follows.

The added value of this study could be carved out more clearly. The three specific goals mentioned are not fully adequate in my opinion Goal 1: Why "to guide the development"? In my view you actually develop such models in the present study. Goal 2: porcine-specific (in general) or specific to an individual pig? Goal 3: "clinical application" is an overstatement in my opinion as most readers would likely assume application to humans The added value of the developed models remains vaguely described. There is mention of supporting the 3R principles but no concrete ways in which this could be achived are mentioned.

We agree with the Reviewer that the added value of the study can be clarified. Following the Reviewer's comment, we modified the last paragraph of the Introduction to better describe the current limitations of preclinical modeling and simulation and the goals of the study. The revised text reads:

"Although *in silico* models are commonly used to support preclinical trials in pigs, many are still largely derived from non-porcine data, which limits mechanistic accuracy, predictive power, and the individualization of key simulation parameters. To overcome these limitations, we propose that specific cardiac conditions can be faithfully reproduced in pigs by tailoring critical modeling parameters, which are identified through extensive multi-modal porcine data and systematically validated with large-scale simulations.

In this context, this work has 4 main objectives: 1) To define a reproducible pipeline for the development and calibration of biventricular (BiV) electrophysiological models in healthy and MI pig hearts by processing cardiac signals and images from *in vivo* and *ex vivo* pig experiments; 2) To identify critical parameters required to reproduce both *in silico* species-specific (porcine) and subject-specific (individual pig) electrical characteristics in healthy and MI conditions; 3) To provide openly available, state-of-the-art BiV porcine models for use in preclinical cardiovascular studies (e.g. drug cardiotoxicity, remuscularization assessment), thereby reducing the reliance on animal experimentation; 4) To show the potential of these experimentally guided *in silico* models for mechanistic analysis of infarction-induced electrocardiogram (ECG) alterations across multiple infarct locations."

Specifically, the main changes in the definition of the specific goals are as follows:

- Goal 1: We rephrased this objective to explicitly define the development of a modeling and simulation pipeline designed for the integration of multi-modal experimental data.
- Goal 2: We clarified that the individualization of key electrophysiological parameters encompasses both species-specific (porcine) and subject-specific (individual pig) contexts.
- Goal 3: A new goal was added to highlight the utility and translational value of the developed models. To support this, we included Section 4.9, "Model usability and preclinical implications", at the end of the Discussion. This section details a specific use case, presents quantitative accuracy metrics and error tolerances, and evaluates whether the models meet these.
- Goal 4: This goal was reframed to move away from direct clinical claims. Instead, it now emphasizes the utility of modeling and simulation as tools for mechanistic analysis of ECG alterations in myocardial infarcts with distinct locations.

I am surprised that there is little ST deviation (elevation or depression) visibile in the infarction ECGs (Fig. 11).

The subtle *in silico* ST deviation responds to the modeling decision of considering the dense scar region of the infarction as an electrical insulator [1]. To accomplish this, the dense scar was extracted from the biventricular model in the infarcted cases, as in [2, 3, 4, 5]. Thus, during the ST segment, all tissue is depolarized, and there are no strong potential gradients across the domain, making the electrocardiographic signal zero in all leads. This modeling decision is supported by the lack of ST-segment deviation observed in the experimental electrocardiogram data from pig 6, which exhibited a chronic infarction in the left circumflex coronary artery. Initial simulations defining the dense scar with the same electrophysiological properties set at the border zone, but with low conductivity and excitability, induced drastic ST deviations that were not observed experimentally. In this line, minimal ST deviation in a chronically infarcted human case was observed in [5]. Similarly, the authors initially defined the dense scar as passive tissue, yielding considerable ECG distortion caused by gradients between the ventricular tissue and the dense scar, which remained at resting potential across the simulations. In order to reproduce their experimental electrocardiogram, the dense scar was defined as an insulator, as in our work. A summary of this modeling considerations is now explicitly highlighted in Section 4.6 of the revised manuscript: "*SZ modeling*: We modeled SZ as an insulator, consistent with previous computational studies [3, 2, 4, 5]. Modeling SZ as passive tissue, as in Connolly et al. [1], can create unrealistically large potential gradients and ST deviations [5], requires the calibration of additional resistance and resting potential parameters, and forces the SZ to act as an electrotonic sink. This sink effect shortens the APD in BZ, necessitating the definition of non-physiologically larger APD in the BZ to compensate."

l. 190: How much beat-to-beat variability did you observe in the activation and APD90 values?

The processing of the optical mapping signals was implemented using a beat-selection criterion to ensure that similar beats were considered, thereby minimizing beat-to-beat variability in depolarization and repolarization measurements. Specifically, only consecutive beats with equal beating rates were selected. Despite the selection process, some residual variability was still observed. To quantify this, we computed the standard deviation of the activation time and APD_{90} values in each pixel (Fig 1). For this evaluation, 11 beats were available for the healthy pig 13, and 4 beats were available for the LAD infarcted pig 10.

In pig 13, the standard deviation of the activation times, in median across pixels, was 20.5 ms and it was 51.5 ms for APD_{90} values, respectively. Pig 10 exhibited a lower median standard deviation of 4.8 ms for AT and 4.6 ms for APD_{90} values; however, as mentioned, fewer beats were available for calculation. Beat-to-beat variability for repolarization was notably higher near the infarction zone in pig 10. In both pigs, higher repolarization variability was observed in regions covered with fat, such as the interventricular septum (see Fig 1). Due to the existence of this regional and global beat-to-beat variability, we determined that reporting the median values for activation times and APD_{90} values provided a more accurate and representative summary of the optically mapped electrophysiological data.

l. 322: "These porcine-specific pig-wise values of LDC and gK1 defined the mean values that were used in cases where personalization was not possible due to the lack of electrophysiological data." Unclear to me

We agree with the Reviewer that this phrase should be clearer. Therefore, we rephrased it to: "As detailed in Table 1, the individualization of these parameters was not feasible for all pigs due to the absence of the necessary experimental electrophysiological data. In these specific cases, the LDC and g_{K1} parameters were instead assigned the mean values calculated from the subset of pigs for which experimental data were available (see, for example, LDC definition in Table 4)".

It is a pity that ECG is only available for two of the pigs. This would be a very valuable resource for model validation.

We agree with the Reviewer that having more ECG data would enhance model validation. However, these are the only ECGs available for these experiments under healthy and infarcted conditions.

Does the need for separate alignment of QRS complexes and T-waves indicate thate AP duration / morphology is not well matched between simulations and experiments?

We acknowledge that reporting separate analyses of QRS complexes and T waves can be misinterpreted as a mismatch between *in silico* and experimental AP duration / morphology. To avoid this, we included the complete ECG waveforms of the three simulated healthy pigs 1-3, together with the complete experimental ECG of pig 13, in supporting S2 Figure. In the manuscript, we added the following text at the end of Section 3.3: "Moreover, good agreement between the experimental and simulated duration of the ST segment was achieved. This agreement can be observed in S2 Figure, which shows the complete simulated and experimental ECG waveforms for healthy pigs 1-3 and pig 13, respectively."

In supporting S2 Figure, good agreement can be qualitatively observed in the alignment of QRS complexes and T waves of all leads between the experiment and the simulations across our pool of three healthy porcine models, suggesting biomimetic *in silico* AP morphology and duration. In some leads, small alignment differences occurred, possibly due to two reasons. The first cause could be the inter-pig variability between the experiment and simulations, as the experiment involved pig 13, and the simulations involved pigs 1-3. In this line, the *in silico* APD heterogeneities were defined from the experimental work of Meijborg [6] and were not adjusted *ad hoc* to replicate the experimental T waves. The second cause could be related to the limitations of our methodology for *in silico* ECG calculation, where no conductivity variations were considered between the heart and the electrodes, as the organs (e.g., lungs) and structures (e.g., ribs) were not included in our modeling. In spite of these small discrepancies, the achieved *in silico* replication of the experimental ECG remains highly accurate, even though no adjustment of the ventricular APD heterogeneities was performed for T-wave

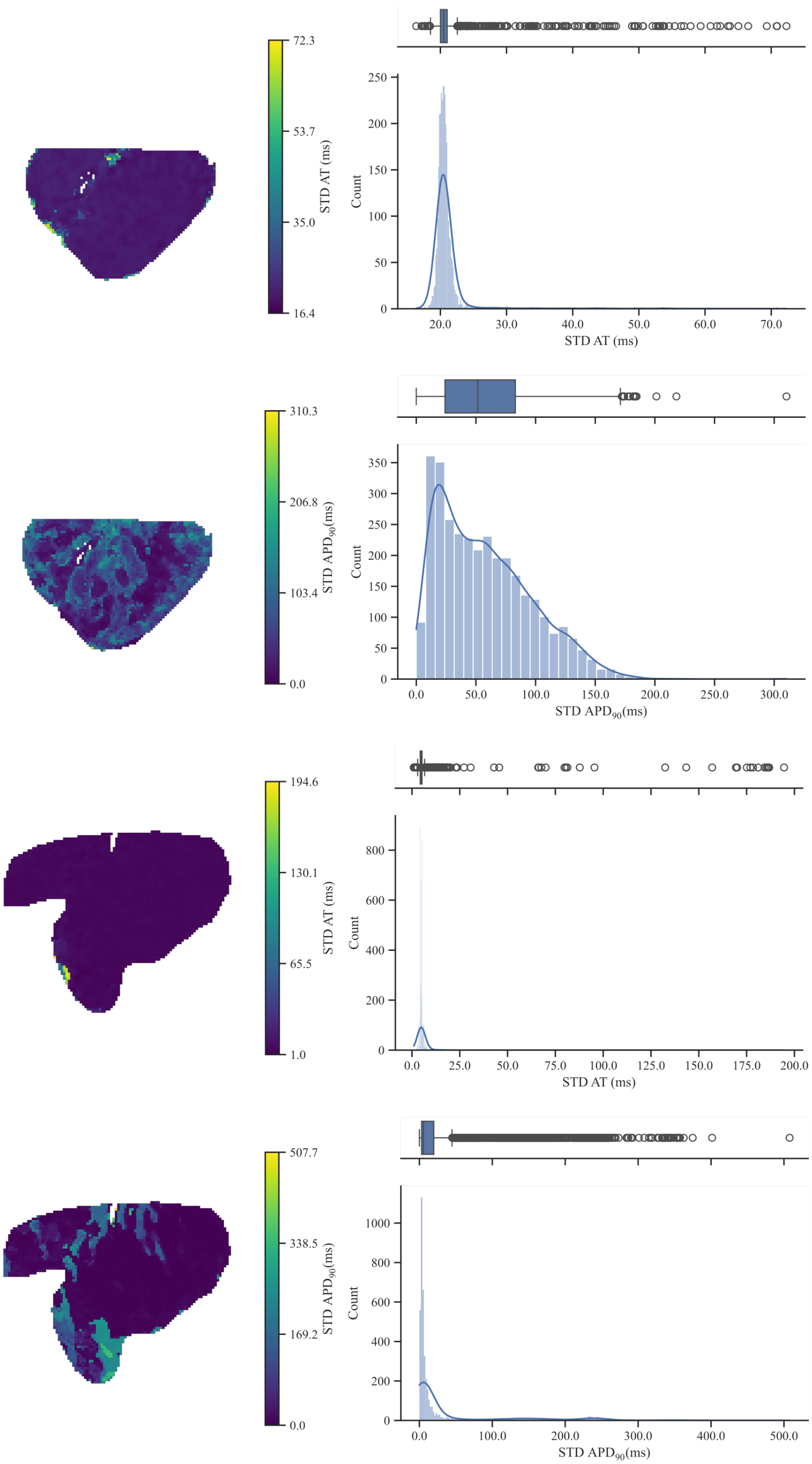

Figure 1: **Beat-to-beat variability in optical mapping data.** Pixel-wise standard deviation maps (left) and corresponding histograms (right) for the calculated activation times and APD_{90} values across recorded beats. Top: results for the healthy pig 13. Bottom: results for the LAD infarcted pig 10.

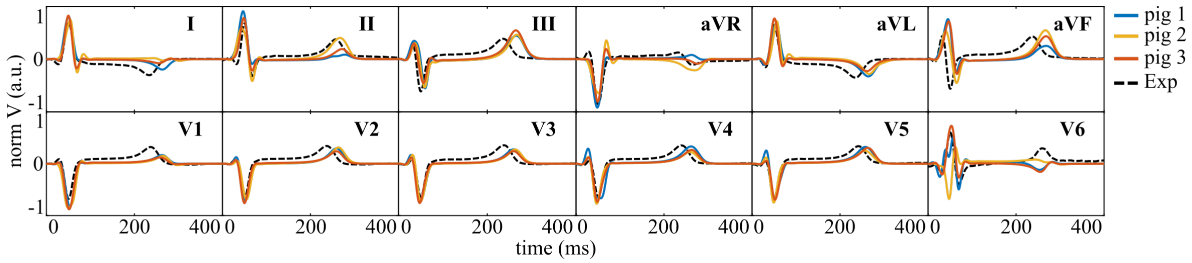

Figure 2: **Simulated and experimental ECGs under healthy conditions.** The complete simulated ECGs obtained for the three healthy pigs 1-3 when using the adjusted CS, APDSH, and s-RBM are shown together with the experimental ECG of pig 13. ECG: Electrocardiogram, CS: Conduction system, APDSH: Action potential duration spatial heterogeneities, s-RBM: Standard rule-based model.

replication. This *in silico* replication outperforms or matches those presented in previous works [5, 7].

Fig. 7: What is the reason for V6 being negative in all simulations whereas it is positive in the experimental recording?

Following the Reviewer's question, we investigated the origin of the negative T wave in lead V6 in all simulations, in contrast to the positive T wave seen in the experiments.

Several computational studies have shed light on how action potential transmural heterogeneities shape the T wave [8, 9]. Specifically, Gima and Rudy [8] *in silico* demonstrated a positive extracellular unipolar potential when the repolarization wave moves away from a virtual recording electrode, which occurs when the epicardium repolarizes earlier than the endocardium. This observation was reiterated in our work, where the precordial unipolar electrodes V1-V5 exhibited positive T waves, as these electrodes saw the endocardium hidden behind the epicardium. Based on experimental recordings, the epicardium of pigs was consistently associated with a shorter APD than the endocardium (see Table 3 in the revised manuscript) [6]. Therefore, we contend that the model's lack of a portion of the ventricular base and outflow tracts, combined with the higher position of the V6 electrode in the apico-basal direction parallel to the interventricular septum, rendered electrode V6 unable to clearly detect a fast-repolarizing epicardium closer than a slow-repolarizing endocardium, which would ultimately yield a positive T wave in simulations, as distinctly observed in the other precordial leads.

To briefly add this reasoning to the manuscript, we modified the second phrase of the second paragraph of Section 4.3:

"Lead V6 remained the most challenging for T-wave matching, likely due to the specific electrode position combined with the incomplete representation of the ventricular base and outflow tracts in the model."

The data acquisition and processing for this study is a major effort. How were the cine CMR data described in section 2.2.1 used? I have the impression that only the end-diastolic state was used.

We thank the Reviewer for acknowledging the data acquisition and processing effort. As pointed out by the Reviewer, the cine CMR data were used to define the biventricular geometry through the segmentation of the end-diastolic images, which we considered the appropriate time point to set the electrophysiological phenomenon preceding the mechanical contraction. Moreover, the end-diastolic images of the cine CMR data were used for the initial determination of the insertions of the left anterior and posterior papillary muscles, which served to generate the conduction system architecture in the healthy pigs.

These two uses are indicated in the revised manuscript, in Section 2.6.1: "Geometries for healthy pigs were derived from end-diastolic cine-CMR, ..." and in Section 2.6.2: "Consistent with experimental studies, the LV bundle branches terminated at papillary muscles, whose insertions were identified from the end-diastolic cine-CMR [10, 11], while the RV bundle ...".

Code and model setups are available, which is comendable. What about the raw data? It seems

that a huge part of the effort is related to data acquisition and not all of them were used, so it would be a pity if others couldn't build on it.

We thank the Reviewer for acknowledging the effort in making models and simulation configurations available. We would like to clarify that every experiment mentioned in Table 1 was directly or indirectly used in the calibration or validation of the modeling and simulation presented herein. In this context, we have extended and refined Section 2.7 "Numerical Simulations" of the revised manuscript. This section now provides a straightforward description of the usage of experimental data: "'Even though complete experimental datasets were not available for every pig, we carefully designed our modeling framework to maximize individualization whenever possible, both at the species-specific level and, when feasible, at the subject-specific level. As shown in Table 4, experimental data were used for input (feeding) and validation of the modeling and simulation:

- *CMRs: In vivo* data were used to individually define the geometry of the 12 BiV models generated: the end-diastolic image from cine-CMR for the 3 healthy cases and the LGE-CMR for the 9 MI (4 LCx and 5 LAD) cases. DW-CMR data were used for subject-specific calibration of the rule-based fiber orientation in healthy cases (DW-CMR from pigs 1-3 in G4) and to define species-specific reductions in electrical conductivity anisotropy in MI cases (DW from pigs 7 and 14 in G5-7).
- *Thoracic magnetic resonance:* Images of pig 6 were used to the define the HERP for pigs in simulation groups assessing the ECG (G2-4 and G6-7).
- *OM:* Mapping data were used exclusively for validation in the G4 simulations, whereas in the G5 simulations they were used for subject-specific model calibration and validation. In G4, *in silico* ATs, depolarization patterns, CVs, and APD_{90} values were validated against their OM-obtained counterparts. In G5, OM measurements of CV and APD_{90} in the BZ were used to calibrate exclusively the values of LDC and g_{K1} in BZ for MI pigs 8-12, while ATs, depolarization patterns, and the values of CV and APD_{90} in the HZ were used for validation. The resulting subject-specific calibration in G5 was used in the following G6-7 simulations for defining average species-specific values of LDC and g_{K1} in BZ for the MI models.
- *EAM:* Mapping data from pig 6 were used to define a subject-specific post-MI activation of the LV in pig 6 at the G6 simulations.
- *ECGs:* The healthy ECG recording from pig 15 was used to validate species-specific CS distributions and repolarization heterogeneities (G2-3), while the MI ECG recording was used to assess ECG replication under post-MI conditions, including species-specific alterations on PMJ spatial distributions or the incorporation of subject-specific activation pattern into the MI model of pig 6 (G6)."

Moreover, the experimental data used here are the result of clearing a larger pool of data that sometimes contained corrupted, low-quality information. Then, following the Reviewer's suggestion, the analyzed optimal data listed in Table 1 will be uploaded to a repository that will soon be openly available through this link `https://doi.org/10.5281/zenodo.18223187`. We have updated the Section "Data availability" accordingly and provided an unrestricted access link with this resubmission to facilitate the Reviewer's evaluation of the data.

The manuscript makes use of so many abbreviations that it is hard to remember all of them, which makes following the results section tedious, e.g. "At small PMJ radii as 0.1 mm, ncPMJ was low (28.5% for be-CS, 10.4% for bi-CS), resulting in limited aPMJ (28% for be-CS, 7% for bi-CS)." The same applies to many figure captions, which are full of abbreviations preventing the reader to interpret the figure without having found and memorized all abbreviations. Figure 10 for example is uninterpretable to me without a list of abbreviations (in mind).

We agree with the Reviewer that there are many abbreviations, which undermine the reading flow and rapid image interpretation. To fix this, we erased abbreviations appearing fewer than eight times in the text, such as the abbreviations involving the source-sink mismatch evaluation pointed out by the Reviewer and the "FA" for fractional anisotropy. Moreover, we indicated in the captions of every

Figure and Table the definitions of the abbreviations present in them.

Further comments: I would advise to avoid the term "personalized" when referring to pigs rather than persons. "Individualized" might be an alternative.

We thank the Reviewer for suggesting this. We have changed the term “personalized” to “individualized” throughout the manuscript.

The Cardiac Electrophysiological Simulation Challenge (CESC10); Camara et al., Prog Biophys Mol Bio 2011 and the CRT-EPIGGY19 https://crt-epiggy19.surge.sh seem worthwhile discussing in the context of this work.

We thank the Reviewer for suggesting this. Indeed, we believe these previous works are highly related to ours and therefore merit discussion.

As these works represent the current state of the art for incorporating multi-modal datasets into preclinical models, we decided to mention them in the Introduction of the revised manuscript as follows:

“Initially, generic models with average values of electrophysiological parameters were used for general-purpose simulations. Advances in computational power, memory capacity, and access to biochemical and imaging data have enabled biophysically detailed mathematical simulations across a wide range of scenarios [12, 13]. For pigs, the CESC10 [14] and CRT-EPiggy19 [15] challenges constituted the first endeavors to provide multi-modal experimental data for individualizing preclinical models and assessing their performance. These subject-specific models have been shown to, for example, reproduce inter-individual variability in the generation of reentrant arrhythmias, outperforming generic models [4]. However, the creation of fully individualized digital twins involves time-consuming and technically complex experiments, often limiting the feasibility of parameter individualization. Current efforts focus on identifying and tailoring key electrophysiological parameters required to accurately replicate specific heart conditions [16, 17, 5, 18, 4, 19].”

Furthermore, in the “Limitations, uncertainties and future work” section, we discuss uncertainties in model definition and the use of overly simplistic or inappropriate numerical settings. In the context of “Modeling complexity” uncertainties, we contrast our approach with the studies emerging from the CESC10 and CRT-EPiggy19 challenges. Specifically, we address differences in the usage of experimental data and in the methodological integration within the modeling and simulation pipeline while offering perspectives on future research. The added text in the “Limitations, uncertainties and future work” section reads:

“*Modeling complexity:* While fine-grained, local variations in APD were used to reproduce experimental ECG and OM data, global conductivities were applied to replicate experimental activation features in both healthy and MI conditions. Previous research stemming from the CESC10 [14] and CRT-EPiggy19 [15] challenges has underscored the necessity of regional CV modeling to improve *in silico* agreement with experimental results. Specifically, Camara et al. [17] demonstrated that phenomenological-Eikonal modeling, when combined with detailed biophysical models, enhances the reproduction of OM-measured paced activation in porcine models using DW-CMR-defined geometries and fiber fields. Furthermore, Albors et al. [19] improved the prediction of EAM activation in left-bundle branch block conditions by partitioning ventricular tissue into regions and optimizing conductivity for each of these regions. Despite our use of global conductivities, our approach successfully replicated depolarization characteristics from both tissue-level OM data and organ-level relevant ECG measurements.

Future work should leverage more granular modeling pipelines that integrate findings from previous computational challenges with novel, species-specific experiments, which are currently lacking in the literature, to further validate and extend our observations while reducing reliance on cross-species and / or cross-individuals parameterization. Despite the mentioned constraints, this study integrates an exceptionally broad range of experimental data. It extends the efforts of previous benchmarks, such as CESC10 [14] and CRT-EPiggy19 [15], by incorporating multi-sequence functional and structural CMR, OM and EAM measurements in sinus rhythm, and clinically relevant ECG data. Ultimately, this integrated pipeline identifies the modeling features critical for replicating ventricular electrophys-

iology across multiple scales in both healthy and infarcted porcine hearts."

l. 140: What are "custom-designed ad hoc containers"?

We agree with the Reviewer's assessment that this section required further clarification. Accordingly, we have revised the text as follows: "At KUL, hearts were arrested in end-diastole, excised with the great vessels, and perfused via the coronary arteries with 1 L of cold cardioplegic solution at a pressure of 100 mmHg, then immersed in the same solution for 1 hour. The great vessels were firmly tied to maintain pressure, and the hearts were placed in a individualized 3D-printed cast filled with cardioplegic solution (left panel of S1 Figure). These custom-designed containers were constructed using the epicardial segmentation of the *in-vivo* cine CMRs [20]. At SERMAS an atrial transseptal puncture was performed to equalize intracavitary volumes. Warm 2% agarose gel solution (≈38 °C) was infused via cannulas in the aorta and pulmonary artery until the ventricular cavities approximated an *in vivo* diastolic state. The heart was positioned within a container of sufficient size to prevent compression and was embedded in warm agarose. Following embedding, the assembly was refrigerated to promote agarose gelation and stabilize the organ, thereby minimizing unwanted myocardial deformation and maintaining structural integrity (right panel of S1 Figure)."

Furthermore, we have included supporting Fig 3 to illustrate the *ex vivo* acquisition process, which includes representative DW-CMR images obtained at both KUL and SERMAS.

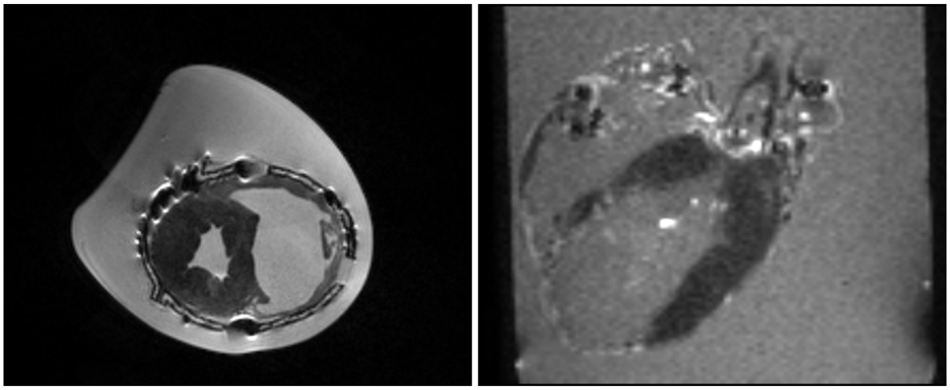

Figure 3: **DW-CMR acquisition protocols.** Representative transverse view of the healthy porcine heart (pig 3) acquired at KUL (left) and coronal view of the LAD-occlusion MI model (pig 14) acquired at SERMAS (right). DW-CMR: Diffusion-weighted cardiac magnetic resonance, KUL: Katholieke Universiteit Leuven, SERMAS: Servicio Madrileño de Salud, LAD: Left anterior descending, MI: Myocardial infarction.

Why did you use the multi-diffusion approach to differentiate the different transmural layer and not also a Laplace solution as for the apico-basal gradient?

We thank the Reviewer for pointing this out. Our reference to the multi-diffusion problem pertains to the definitions provided in Perotti et al. [9], where the steady-state diffusion (Laplace) equation was solved multiple times with different boundary conditions to define the transmural segmentation of the ventricles. Thus, by multi-diffusion, we were referring to the combination of multiple Laplace solutions. To clarify this, the first phrase of the second paragraph of Section 2.6.5 in the revised manuscript now reads: "To include transmural heterogeneities, the steady-state diffusion (Laplace) equation was solved multiple times with different boundary conditions [9] to accurately segment the BiV mesh transmurally (orange dashed region in Fig 3c)."

l. 380: Is there experimental evidence that spatial APD heterogeneity in pigs is due to differential expression of IK1?

Following the Reviewer's comment, we performed a more extensive literature review regarding the role of I_{K1} and APD heterogeneity in pigs.

Experimental data on APD heterogeneity in the porcine ventricle are limited and generally depict only minimal transmural APD differences. Some studies found no statistically significant differences in transmural APD [21, 22], while others reported small but significant differences [6]. Crucially, none of these experimental works effectively demonstrated a direct correlation between the observed APD variations and I_{K1} expression.

The evidence in this regard remains indirect: the downregulation of I_{K1} is known to play a major role in APD prolongation in the failing heart across species [23]. The computational study by Gaur et al. [24] has explicitly suggested the importance of I_{K1} in defining porcine APD. This computational finding by Gaur et al. [24], combined with the known I_{K1} alterations in the failing heart [23], supports our modeling decision to tune APD, specifically in the MI cases, via modulation of g_{K1}. In line with this, the minimal APD heterogeneities in the healthy case were also implemented through variations in the same conductance g_{K1}, resulting in an improved *in silico* reproduction of the experimental T waves.

Taken together, our modeling outcomes, combined with those of Gaur et al. [24], support the hypothesis that I_{K1} modulates APD in both pathological (MI) and likely healthy porcine hearts. As this specific mechanistic result remains purely computational and its direct experimental confirmation is still insufficient, we have modified the end of Section 4.3 in the revised manuscript, which now reads:

"To our knowledge, this is the first computational study to reproduce experimentally observed porcine APDSH, achieved here by modifying g_{K1} in the Gaur et al. [24] model. This *in silico* mechanistic insight suggests that I_{K1} plays a more dominant role in modulating the porcine APD than other K^+-related currents typically emphasized in human models. This finding is consistent with experimental evidence showing that pigs exhibit high sensitivity to proarrhythmic effects induced by I_{K1} blockade [23], while remaining largely insensitive to I_{Kr} inhibition [25]. However, because quantitative porcine-specific data on regional ionic current densities remain limited, further experimental studies are warranted to determine whether I_{K1} modulation more accurately reflects the underlying porcine repolarization gradients."

Table 3 misses units

We thank the Reviewer for pointing this out. We have added the units to Table 3, which is now:

Table 1: **Definition of repolarization heterogeneities.** The target (T) and simulated (S) APD_{90} values (at 650 ms cycle length) are shown together with the g_{K1} (x10) values leading to the corresponding simulated APD_{90}. B: Base, M: Middle, A: Apex, En: Endo, Ep: Epi, g_{K1}: Maximum conductance of the inward rectifier K^+ current.

	**B**			**M**			**A**		
	T (ms)	S (ms)	g_{K1} (mS/µF)	T (ms)	S (ms)	g_{K1} (mS/µF)	T (ms)	S (ms)	g_{K1} (mS/µF)
**En**	213.9	213.9	1.49	222.7	222.4	1.39	218.3	218.1	1.44
**M**	204.8	204.5	1.61	213.2	213.2	1.5	208.9	208.5	1.56
**Ep**	199.0	199.4	1.68	207.2	207.3	1.57	203.1	203.1	1.63

l. 392: "80-µA/cm2" -¿ "80 µA/cm2"?

We thank the Reviewer for pointing this out. We have made the correction at Section 2.7.

l. 398: I thought optical mapping data were used to parametrize APD, how can it be used for validation then? Section 3.5 also mentions that optical mapping data were used to calibrate border zone electrophysiology. Please clarify.

We agree with the Reviewer that the description of the use of optical mapping data in the modeling and simulation was unclear. Optical mapping data were used exclusively for validation in the G4 simulations, whereas they were used for both model calibration and validation in the G5 simulations.

In the G4 simulations, activation times, depolarization patterns, conduction velocities, and action potential duration at 90% repolarization values obtained *in silico* were validated against experimental optical mapping data.

In the G5 simulations, experimentally measured conduction velocities and action potential duration at 90% repolarization values from optical mapping of the border zone were used to calibrate **only** the border zone conductivity and g_{K1} in the MI cases. Activation times and depolarization patterns derived from optical mapping of the entire anterior cardiac surface (encompassing both healthy and infarcted regions) were then used to validate the *in silico* simulations. In addition, conduction velocities and action potential duration at 90% repolarization values measured in the healthy regions from optical mapping were used to validate the corresponding quantities in the healthy regions of the MI simulations.

To clarify this in the manuscript, we modified section 2.7 "Numerical simulations":

"Even though complete experimental datasets were not available for every pig, we carefully designed our modeling framework to maximize individualization whenever possible, both at the species-specific level and, when feasible, at the subject-specific level. As shown in Table 2, experimental data were used for input (feeding) and validation of the modeling and simulation:

- *CMRs: In vivo* data were used to individually define the geometry of the 12 BiV models generated: the end-diastolic image from cine-CMR for the 3 healthy cases and the LGE-CMR for the 9 MI (4 LCx and 5 LAD) cases. DW-CMR data were used for subject-specific calibration of the rule-based fiber orientation in healthy cases (DW-CMR from pigs 1-3 in G4) and to define species-specific reductions in electrical conductivity anisotropy in MI cases (DW from pigs 7 and 14 in G5-7).
- *Thoracic magnetic resonance:* Images of pig 6 were used to the define the HERP for pigs in simulation groups assessing the ECG (G2-4 and G6-7).
- *OM:* Mapping data were used exclusively for validation in the G4 simulations, whereas in the G5 simulations they were used for subject-specific model calibration and validation. In G4, *in silico* ATs, depolarization patterns, CVs, and APD_{90} values were validated against their OM-obtained counterparts. In G5, OM measurements of CV and APD_{90} in the BZ were used to calibrate exclusively the values of LDC and g_{K1} in BZ for MI pigs 8-12, while ATs, depolarization patterns, and the values of CV and APD_{90} in the HZ were used for validation. The resulting subject-specific calibration in G5 was used in the following G6-7 simulations for defining average species-specific values of LDC and g_{K1} in BZ for the MI models.
- *EAM:* Mapping data from pig 6 were used to define a subject-specific post-MI activation of the LV in pig 6 at the G6 simulations.
- *ECGs:* The healthy ECG recording from pig 15 was used to validate species-specific CS distributions and repolarization heterogeneities (G2-3), while the MI ECG recording was used to assess ECG replication under post-MI conditions, including species-specific alterations on PMJ spatial distributions or the incorporation of subject-specific activation pattern into the MI model of pig 6 (G6)."

Fig. 6b: It is not obvious at first sight why there is only 1 "exp" trace if there are 3 pigs. From Table 1 it becomes clear that this is not a 1:1 comparison. This should be highlighted in the figure caption.

We agree with the Reviewer that the description of Fig 6 can be clearer by highlighting that experimental versus simulated ECG comparisons are not 1:1. The revised caption for Fig 6 is:

"**Effect of CS distribution and HERP on simulated QRS complexes.** a- Simulated QRS complexes for pig 3 when different HERP and CS distributions were used are depicted with the experimental QRS complexes from pig 15 (Exp, black dashed line) . Semi-transparent and solid lines represent the results when the b-HERP and a-HERP were used, respectively. b- Simulated QRS complexes obtained with a-HERP and ap-CS for pigs 1-3 are depicted with the experimental QRS complexes from pig 15 (Exp, black dashed line). c- Simulated versus experimental mean QRS similarity obtained for pigs 1-3 when different HERP and CS distributions were employed. d- Mean QRS similarity for pigs 1-3 for simulations with ap-e-CS and ap-CS distributions. CS: Conduction system, bi-CS: Baseline intramyocardial CS, rvs-CS: bi-CS with Purkinje in the septal wall of the right ventricle, ap-CS: rvs-CS with the end of the left bundle branches closer to the apex of the left ventricle, ap-e-CS: ap-CS with endocardial distribution, b/a-HERP: Baseline/adjusted heart-electrode relative

Table 2: **Simulation groups.** Parameters in square brackets were varied in the simulation group for evaluation. Average LDC ($\overline{\text{LDC}}$) and g_{K1} ($\overline{g_{K1}}$) values were defined as the mean of the OM-adjusted LDC^i and g^i_{K1} values at the BZ of pigs 8-12, therefore, i=8,...,12. PMJ: Purkinje-muscular junction, PMJr: PMJ radius, b-LDC: Baseline longitudinal diffusion coefficient, g_{K1}: Maximum conductance of the inward rectifier K^+ current, OM: Optical mapping, MR: Magnetic resonance, LGE: Late gadolinium-enhanced, DW: Diffusion-weighted, T: Thoracic, CS: Conduction system, bi-CS: Baseline intramyocardial CS, rvs-CS: bi-CS with Purkinje in the septal wall of the right ventricle, ap-CS: rvs-CS with the end of the left bundle branches closer to the apex of the left ventricle, ap-e-CS: ap-CS with endocardial distribution, mi-CS: ap-CS with altered spatial distribution of Purkinje fibers following infarction, e/i-EAM: Endocardial/intramyocardial activation based on the electroanatomical mapping, s/dw-RBM: Standard/diffusion-weighted rule-based model, b/t/c-APDSH: Baseline/transmural/transmural-apicobasal action potential duration spatial heterogeneities, HZ: Healthy zone, BZ: Border zone, b/a-HERP: Baseline/adjusted heart-electrode relative position, ECG: Electrocardiogram.

**Group** (pig)	**Input** (pig)	**Activation**	**PMJr** (mm)	**RBM**	**APDSH**	**LDC**	**HERP**	**Validation** (pig)
**G1** (1)	cine-MR (1)	[be-CS, bi-CS]	[0.1-2]	s-	b-	b-	-	-
**G2** (1-3)	cine-MR (1-3)<br>T-MR (6)	[bi-CS, rvs-CS,<br>ap-CS, ap-e-CS]	0.5	s-	b-	b-	[b-, a-]	ECG (15)
**G3** (1-3)	cine-MR (1-3)<br>T-MR (6)	ap-CS	0.5	s-	[b-, t-, c-]	b-	a-	ECG (15)
**G4** (1-3)	cine-MR (1-3)<br>DW-MR (1-3)<br>T-MR (6)	ap-CS	0.5	[s-, dw-]	c-	b-	a-	ECG (15)<br>OM (13)
**G5** (8-12)	LGE-MR (8-12)<br>OM (8-12)<br>DW-MR (7,14)	ap-CS	0.5	s-	HZ: c-<br>BZ: g^i_{K1}	HZ: b-<br>BZ: LDC^i	-	OM (8-12)
**G6** (6)	LGE-MR (6)<br>EAM (6)<br>DW-MR (7,14)<br>OM (8-12)<br>T-MR (6)	[ap-CS, mi-CS,<br>e-EAM, i-EAM]	0.5	s-	HZ: c-<br>BZ: $\overline{g_{K1}}$	HZ: b-<br>BZ: $\overline{\text{LDC}}$	b-	ECG (6)
**G7** (4-12)	LGE-MR (4-12)<br>DW-MR (7,14)<br>OM (8-12)<br>T-MR (6)	ap-CS	0.5	s-	HZ: c-<br>BZ: $\overline{g_{K1}}$	HZ: b-<br>BZ: $\overline{\text{LDC}}$	b-	-

position."

Fig. 9 should clearly indicate the scar region (which should not be activated at all as I understand).

Following the Reviewer's comment, we added the model's anterior view for the two pigs, depicting the myocardial infarction (border zone plus scar zone). The revised figure is:

Please note that in the two pigs, the non-excitable scar zone is not transmural and, consequently, it does not appear in the anterior view of the models, except for a small region in pig 9. For this reason, almost all the anterior views of the models are activated (the small scar zone region for pig 9 has no activation time). In addition to this information, the complete volumetric MI models can be seen at the bottom of Fig 11, with their infarct regions depicted.

"Personalized fiber orientation" can be misleading as you did not personalize the entire fiber field but only one endocardial and one epicardial angle per ventricle as I understand.

We acknowledge the Reviewer's concern regarding the potential interpretation of "personalized fiber orientation" as strong localized individualization. To mitigate this, we have amended the term throughout the revised manuscript to "subject-specific fiber orientation calibration".

At the same time, we believe that our approach constitutes a critical level of individualization. While the adjustment was limited to the endocardial and epicardial angles for each ventricle, these two parameters govern the entire rule-based fiber field generated within our model. Critically, these

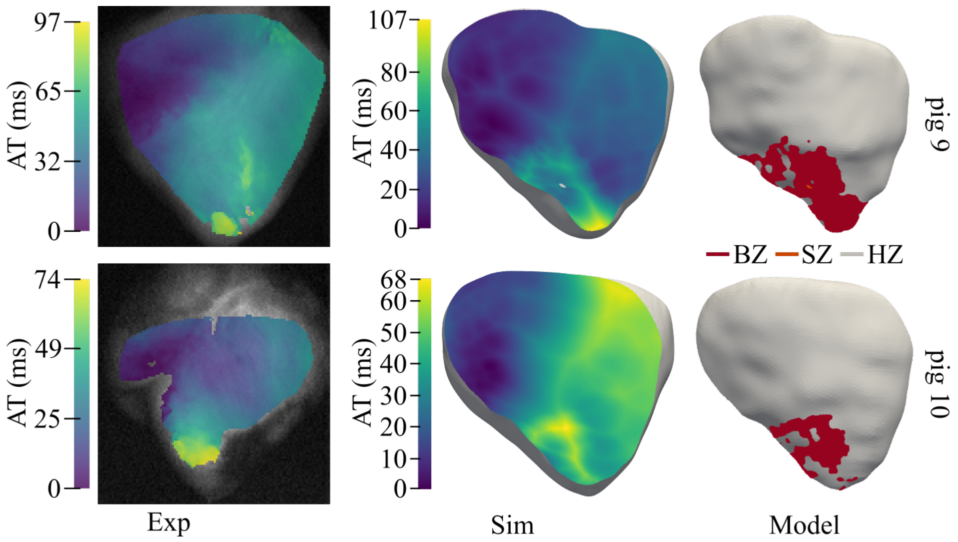

Figure 4: **Experimental and simulated activation after MI.** Experimental (left) and simulated (middle) AT maps of the anterior view for two MI pigs with LAD occlusion. The anterior view of the model for the two pigs is shown at the right side. AT: Activation time, MI: Myocardial Infarction, LAD: Left anterior descending, BZ: Border zone, SZ: Scar zone, HZ: Healthy zone.

calibrated angles were specifically selected to achieve a complete fiber architecture that best matched the diffusion-weighted imaging data across the entire ventricular wall, not just at the boundary layers. As explicitly stated in the Methods, this calibration was a necessary step, as the direct application of the diffusion-derived fiber field was not feasible due to heart deformation for the imaging experiment. Thus, our process involves feeding the subject's diffusion data to calibrate the key global parameters of a rule-based model, which we believe justifies the emphasis on subject-specific calibration.

Reviewer 2:

This manuscript presents an ambitious whole-organ porcine digital-twin electrophysiology framework informed by high-quality imaging and electrophysiological measurements. The integration of fiber architecture, infarct structure, repolarization heterogeneity, and anatomical conduction system (CS) modeling is a strength, and the authors have assembled an exceptional experimental dataset from intact organs. However, several methodological details require clarification, and some modeling assumptions rely on human or canine literature rather than porcine-specific measurements. The manuscript would benefit from clearer justification of those modeling choices, additional methodological transparency, and discussion of model validation requirements for future clinical translation.

We are grateful to the Reviewer for the revisions and for the helpful and constructive comments, which we have addressed as follows.

Abstract
- The similarity metric reported as: "Accurate CS and repolarization heterogeneities reproduced depolarization (0.76 QRS similarity) and repolarization (0.74 T-wave similarity) patterns." should specify the calculation method (Pearson correlation). Without this information, the interpretation of the similarity values is unclear.

We agree with the Reviewer that specifying the calculation methodology of the similarity enhances the interpretation of the results. Thus, in the abstract of the revised manuscript, we have replaced the word "similarity" with "Pearson correlation".

Scope and Framing of the Study
- The current framing presents the work largely as a modeling workflow rather than addressing

either:

* a biological hypothesis, or

* an engineering performance target.

As written, the contribution sits between those categories. Clarifying the intended purpose of the model (mechanistic exploration, validation framework, clinical translation tool, or proof-of-feasibility) would help position the work appropriately.

We thank the Reviewer for the opportunity to clarify the intended purpose of our work. To accomplish this, we have rephrased the last paragraph of the Introduction as follows:

"Although *in silico* models are commonly used to support preclinical trials in pigs, many are still largely derived from non-porcine data, which limits mechanistic accuracy, predictive power, and the individualization of key simulation parameters. To overcome these limitations, we propose that specific cardiac conditions can be faithfully reproduced in pigs by tailoring critical modeling parameters, which are identified through extensive multi-modal porcine data and systematically validated with large-scale simulations.

In this context, this work has 4 main objectives: 1) To define a reproducible pipeline for the development and calibration of biventricular (BiV) electrophysiological models in healthy and MI pig hearts by processing cardiac signals and images from *in vivo* and *ex vivo* pig experiments; 2) To identify critical parameters required to reproduce both *in silico* species-specific (porcine) and subject-specific (individual pig) electrical characteristics in healthy and MI conditions; 3) To provide openly available, state-of-the-art BiV porcine models for use in preclinical cardiovascular studies (e.g. drug cardiotoxicity, remuscularization assessment), thereby reducing the reliance on animal experimentation; 4) To show the potential of these experimentally guided *in silico* models for mechanistic analysis of infarction-induced electrocardiogram (ECG) alterations across multiple infarct locations."

In response to the Reviewer's comment, our work defines a pipeline for the experimentally guided creation of porcine cardiac digital twins (Objective 1) and provides validated porcine biventricular models under healthy and MI conditions (Objective 3). The techniques and models presented enable mechanistic exploration of cardiac conditions (Objective 2) and demonstrate the feasibility for preclinical translation of computational electrophysiology (Objective 4).

Data

- Table 1 presents highly heterogeneous sample characteristics. The authors should comment on whether this variability was expected, and whether it impacts personalization steps or parameter calibration. It was not clear to me how data was combined (or not) to form a digital twin of a specific pig. Or is this more a model of a representative pig?

We thank the Reviewer for this question. The variability in the experimental data available for each pig was expected, as some experimental acquisition methods were incompatible with others (e.g., *ex vivo* DW-CMR versus optical mapping), and some procedures were specific to one of the two centers (e.g., LAD-MI induction and optical mapping were performed at SERMAS, whereas LCx-MI induction and EAM were performed at KUL). Naturally, this variability impacts the generation of fully subject-specific models, as only a limited set of experimental measurements is typically available for each subject due to the cost and complexity of data acquisition. In addition, certain features are often impractical to individualize (e.g., the accurate anatomy of the conduction system).

To address these limitations, we carefully defined our modeling framework to maximize individualization whenever possible, both at the species-specific level and, when feasible, at the subject-specific level. This clarification is summarized in Section 4.8, where we discuss limitations on cross-subject data:

"*Cross-subject data:* Not all experimental modalities were available here for each animal under both healthy and MI conditions, partly because some methods are incompatible (e.g., *ex vivo* DW-CMR versus OM) or center-specific (LAD-MI and OM were performed at SERMAS, and LCx-MI and EAM were conducted at KUL). Consequently, the extent of model individualization differed among subjects. When subject-specific data were unavailable, population-average values were used. This strategy is common in high-fidelity *in silico* studies, given the complexity and cost of full multi-modal datasets [16, 14, 15]."

In this regard, and to answer the Reviewer's question about how data was combined to form a

digital twin, we added an "Input" column to Table 2 (Table 4 in the revised manuscript). This column explicitly indicates the experimental modalities and corresponding pigs used to generate each model within each simulation group. Moreover, we reordered and extended Section 2.7:

"Even though complete experimental datasets were not available for every pig, we carefully designed our modeling framework to maximize individualization whenever possible, both at the species-specific level and, when feasible, at the subject-specific level. As shown in Table 2, experimental data were used for input (feeding) and validation of the modeling and simulation:

- *CMRs: In vivo* data were used to individually define the geometry of the 12 BiV models generated: the end-diastolic image from cine-CMR for the 3 healthy cases and the LGE-CMR for the 9 MI (4 LCx and 5 LAD) cases. DW-CMR data were used for subject-specific calibration of the rule-based fiber orientation in healthy cases (DW-CMR from pigs 1-3 in G4) and to define species-specific reductions in electrical conductivity anisotropy in MI cases (DW from pigs 7 and 14 in G5-7).
- *Thoracic magnetic resonance:* Images of pig 6 were used to the define the HERP for pigs in simulation groups assessing the ECG (G2-4 and G6-7).
- *OM:* Mapping data were used exclusively for validation in the G4 simulations, whereas in the G5 simulations they were used for subject-specific model calibration and validation. In G4, *in silico* ATs, depolarization patterns, CVs, and APD_{90} values were validated against their OM-obtained counterparts. In G5, OM measurements of CV and APD_{90} in the BZ were used to calibrate exclusively the values of LDC and g_{K1} in BZ for MI pigs 8-12, while ATs, depolarization patterns, and the values of CV and APD_{90} in the HZ were used for validation. The resulting subject-specific calibration in G5 was used in the following G6-7 simulations for defining average species-specific values of LDC and g_{K1} in BZ for the MI models.
- *EAM:* Mapping data from pig 6 were used to define a subject-specific post-MI activation of the LV in pig 6 at the G6 simulations.
- *ECGs:* The healthy ECG recording from pig 15 was used to validate species-specific CS distributions and repolarization heterogeneities (G2-3), while the MI ECG recording was used to assess ECG replication under post-MI conditions, including species-specific alterations on PMJ spatial distributions or the incorporation of subject-specific activation pattern into the MI model of pig 6 (G6)."

Anatomical Model

- The mesh excludes the base of the heart. As the base is often the last region activated, it is unclear how this omission may affect the predicted QRS duration or conduction velocity calibration. Clarifying whether conduction was artificially slowed to compensate, or whether truncation explains QRS morphology mismatches, would strengthen the modeling justification.

We thank the Reviewer for this inquiry regarding the truncation of the models in the base. While the basal region is not fully represented, only a limited portion is missing, corresponding to one basal slice and the outflow tract slice, which were not included in the modeling. Considering the cine-CMR and LGE-CMR resolution (see Sections 2.2.1 and 2.2.2), we estimate that the relatively small excluded region corresponds to a thickness of at most 12 mm of the basal tissue. In addition, we would like to clarify that the conduction was not slowed, neither in the tissue nor in the CS, as can be seen from the CV results for the CS (200 cm/s) mentioned in Section 2.6.3 and for the tissue, which was in good agreement and even somewhat higher than the experimental values obtained by OM (Section 3.4).

In this context, we were able to accurately reproduce healthy QRS durations and morphologies in almost all ECG leads (see Fig 6b). This outcome can be explained by our modeling decisions regarding the distribution of PMJs along the endocardium, which for the baseline CS was:

"PMJs were distributed throughout both ventricles, except for the basal area of the LV and the RV, and the septal and posterior regions of the RV, where activation was known to be delayed or initiated by the activation of surrounding tissue [26]."

Nevertheless, the simulated QRS (Fig 6b) and T wave (Fig 7a) in lead V6 lead poorly resembled the experimental ones. We expect that incorporating the missing basal tissue would improve this

correspondence, as lead V6 is positioned in close proximity to this excluded region. Our rationale regarding the T-wave morphology is the following: Several computational studies have shed light on how action potential transmural heterogeneities shape the T wave [8, 9]. Specifically, Gima and Rudy [8] *in silico* demonstrated a positive extracellular unipolar potential when the repolarization wave moves away from a virtual recording electrode, which occurs when the epicardium repolarizes earlier than the endocardium. This observation was reiterated in our work, where the precordial unipolar electrodes V1-V5 exhibited positive T waves, as these electrodes saw the endocardium hidden behind the epicardium. Based on experimental recordings, the epicardium of pigs was consistently associated with a shorter APD than the endocardium (see Table 3 in the revised manuscript) [6]. Therefore, we contend that the model's lack of a portion of the ventricular base and outflow tracts, combined with the higher position of the V6 electrode in the apico-basal direction parallel to the interventricular septum, rendered electrode V6 unable to clearly detect a fast-repolarizing epicardium closer than a slow-repolarizing endocardium, which would ultimately yield a positive T wave in simulations, as distinctly observed in the other precordial leads.

Furthermore, we have included statements regarding the absence of the base affecting healthy ECG replication in the revised manuscript. In section 4.2 of the revised manuscript, we added the following text in relation to the QRS complex:

"Nonetheless, residual mismatches remained in leads II, III, and aVF, while lead V6 remained challenging to match as its position corresponds to the septal base, a region lacking in our model. This anatomical discrepancy makes V6 highly sensitive to even minor shifts in electrode or heart positioning."

In Section 4.3, we added the following text for the T-wave:

"Lead V6 remained the most challenging for T-wave matching, likely due to the specific electrode position combined with the incomplete representation of the ventricular base and outflow tracts in the model."

Segmentation and Image Processing

- The statement: "In MI cases, AZ and HZ were manually identified from the automatic deep learning–based segmentations..." appears contradictory. It would help to clarify whether the method is:

* fully manual,
* fully automated, or
* automated with manual correction.

We agree with the Reviewer that this sentence required clarification. For this reason, we have modified and extended this description in Section 2.6.1. In the revised manuscript, the text now reads:

"In MI cases, the BiV domain was reconstructed using a semi-automatic approach. Initial definitions of AZ and HZ, corresponding to high and low voxel intensities, respectively, were obtained from the automatic deep learning–based segmentations of LGE images (Section 2.2.2). These LV segmentations were manually extended to the RV to generate BiV structures. Once a BiV segmentation with HZ and AZ was defined, AZ was subdivided into BZ and SZ using the full-width half-maximum method [27]."

Electrophysiology Model Construction

A number of model components originate from non-porcine literature:

Component Source species

Sodium channel (gNa) remodeling Human [11,16] and canine [48]

Stewart Purkinje model Human [44]

Conduction system conductivity Human estimates [43]

Border zone remodeling Human and canine

Transmural repolarization gradients Human [51,52]

Because these choices may influence behaviour, the authors should comment on:

- expected inter-species electrophysiological differences,

- whether porcine-specific measurements are planned or needed,

- and whether substitution of human/canine parameters may bias conduction or repolarization dynamics.

We thank the Reviewer for raising this important point. To address the Reviewer's comment, we extended the Section "Limitations, uncertainties and future work" to discuss constraints of parameter calibration and the results of a sensitivity analysis performed for these cross-species parameters. The added text is as follows:

"*Cross-species data:* Although pigs are widely regarded as a robust translational model for human cardiac electrophysiology, relevant inter-species differences remain. In particular, quantitative porcine data of APDSH ratios, CS electrophysiology, and BZ ionic remodeling remain scarce. Consequently, several parameters were adapted from human or canine studies that reproduce qualitative electrophysiological behaviors characteristic of large mammalian hearts [28, 29, 30, 26, 31, 32, 33, 3, 5]. To assess the impact of this limitation, a sensitivity analysis was performed on APDSH ratios (S4 Figure), myocardial (S5 Figure) and CS (S6 Figure) conductivities, as well as post-MI I_{Na} remodeling (S7 Figure). At the ECG level, only minor variations were observed, including slightly shorter or longer QRS complex durations for LDC variations in the CS and myocardium, and small S-wave deflections associated with increased BZ excitability remodeling. At the tissue level, electrophysiological markers were insensitive to variations in APDSH ratios. Modifying CS and myocardial LDC by $\pm 20\%$ changed maximum AT and median CV by less than 10%. In contrast, $\pm 6\%$ variations in BZ g_{Na} resulted in changes of approximately 10% in activation-related metrics, underscoring the need for improved calibration of this parameter using porcine-specific post-MI data to further reduce output uncertainty. Overall, this sensitivity analysis suggests that most cross-species parameter choices do not compromise model robustness; however, certain parameters may introduce bias. To mitigate this effect, key outputs (AT, CV, APD_{90}, depolarization patterns, and ECG morphology) were calibrated against porcine experimental OM and ECG data, ensuring physiological consistency with the porcine phenotype."

Regarding future directions, although our current findings indicate that the chosen parameter substitutions do not introduce qualitative bias in the simulated activation or repolarization sequences, we recognize that porcine-specific measurements, particularly of I_{Na} kinetics in the border zone, would be highly valuable for further improving model fidelity. Overall, by combining cross-species parameters with rigorous subject-specific calibration against experimental OM and ECG data, we consider the present model to be a reliable representation of the porcine phenotype, appropriate for the objectives of this study and the use cases outlined in Section 4.9.

APD Heterogeneity Strategy

- A sensitivity analysis on the imposed apex–base and transmural heterogeneity would be valuable, particularly because some transitions are implemented as discrete step changes rather than smooth gradients. It is unclear whether such abrupt transitions are physiologically plausible.

- Prior work (e.g., Camps J et al. Medical Image Analysis) implemented APD heterogeneity using IKs modulation in human models. Qian Nat Card Res used both Iks and Ikr in 3000+ cases. Gillete used Ikr.

- Discussion is warranted on whether:

* Kgr/gks modulation reflects species differences,

* a difference in underlying ion-channel experimental evidence,

* or a constraint of the specific porcine cellular model used.

Following the Reviewer's comment, we have addressed the concerns regarding the physiological plausibility of APD transitions and the impact of the APDSH modeling.

- *Smooth gradients at the interface of APD regions:* While the APD regions (endo, mid, epi, and apex-base) are defined as discrete zones in the model setup, the resulting computational APD distribution at the interfaces between these regions varies gradually. Figure 5 shows smooth APD gradients between regions with different APD definitions, such as at the CS-myocardium and the apicobasal regions of the epicardium. This smoothing effect occurs due to the diffusive coupling inherent in the monodomain equation, as previously characterized in another work using the same solver [34]. This transition mimics the gradual gradients observed experimentally [35], ensuring that the simulated repolarization is not characterized by non-physiological step-discontinuities.

- *Sensitivity analysis and data provenance:* As suggested by the Reviewer, we performed a sensitivity analysis on the APDSH transmural and apicobasal ratios. This was particularly necessary because these ratios were initially defined using human reports and empirical estimations due to the

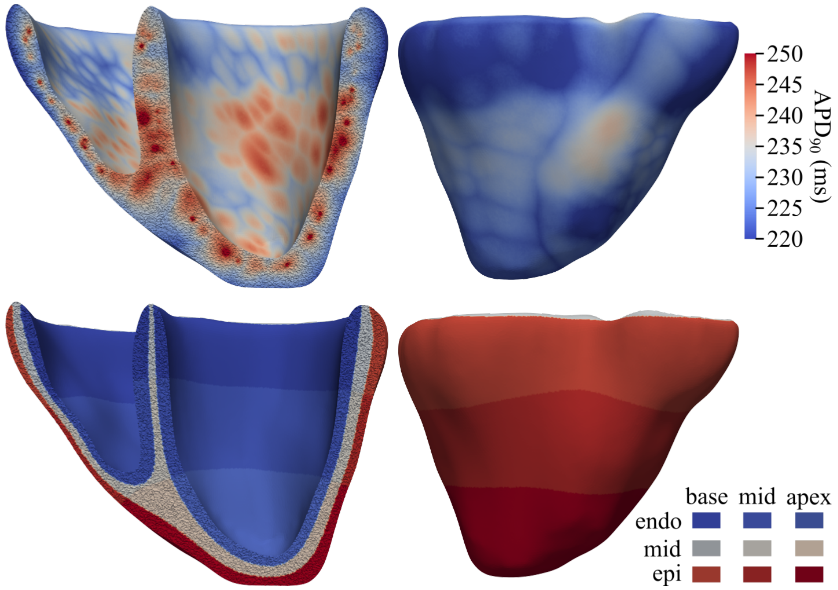

Figure 5: **Simulated APD distributions for pig 1.** Front (coronal cut, left) and back (right) views of the simulated APD values (top) and corresponding anatomical APDSH regions (bottom). The high APD of the CS modulates the surrounding myocardium, shifting the local APD toward higher values near the PMJs. As shown in the posterior view (right), no discrete APD discontinuities are present. For instance, a smooth transition can be observed between the base (lowest epicardial APD) and the mid-ventricular (highest epicardial APD) epicardium.

current lack of specific porcine experimental data for these regional proportions. As detailed in the supporting Fig 6, twelve APDSH configurations were evaluated. Our results demonstrate that variations of ±10% in the size of the APDSH regions yielded consistent T-wave morphologies and minimal amplitude fluctuations, primarily in leads II, III, aVF, and V4–V6. Moreover, negligible variations in terms of electrophysiological outputs at the tissue-level were observed (Fig 6b). Altogether, these results suggest that the model is robust to minor deviations in these parameters, mitigating the bias introduced by using non-porcine sources.

The results of this sensitivity analysis on the APDSH modeling have been incorporated into Section 4.3 of the revised manuscript:

"The proportions of transmural and apicobasal regions were defined based on human-related reports [28, 29] and empirical estimations, respectively. To assess how the limited availability of porcine-specific experimental data on the size of these regions might affect our results, we performed a sensitivity analysis of transmural and apicobasal APDSH distributions on *in silico* repolarization. As shown in S4 Figure, ±10% variations in layer sizes produced consistent T-wave morphologies and median APD_{90} values, indicating that the model is robust to small deviations in these parameters."

Also, the results are discussed in Section 4.8 involving cross-species data:

"To assess the impact of this limitation, a sensitivity analysis was performed on APDSH ratios (S4 Figure), myocardial (S5 Figure) and CS (S6 Figure) conductivities, as well as post-MI I_{Na} remodeling (S7 Figure). At the ECG level, only minor variations were observed, including slightly shorter or longer QRS complex durations for LDC variations in the CS and myocardium, and small S-wave deflections associated with increased BZ excitability remodeling. At the tissue level, electrophysiological markers were insensitive to variations in APDSH ratios. Modifying CS and myocardial LDC by ±20% changed maximum AT and median CV by less than 10%. In contrast, ±6% variations in BZ g_{Na} resulted in changes of approximately 10% in activation-related metrics, underscoring the need for improved calibration of this parameter using porcine-specific post-MI data to further reduce output uncertainty. Overall, this sensitivity analysis suggests that most cross-species parameter choices do not compromise model robustness; however, certain parameters may introduce bias. To mitigate this effect, key outputs (AT, CV, APD_{90}, depolarization patterns, and ECG morphology) were calibrated against porcine experimental OM and ECG data, ensuring physiological consistency with the porcine phenotype."

a)

	Transmural			Longitudinal		
	endo	mid	epi	apex	mid	base
baseline	40	35	25	17	35	48
APDSHL1	40	35	25	22	40	38
APDSHL2	40	35	25	12	30	58
APDSHL3	40	35	25	22	25	53
APDSHL4	40	35	25	12	45	43
APDSHL5	40	35	25	7	40	53
APDSHL6	40	35	25	27	30	43
APDSHT1	30	40	30	17	35	48
APDSHT2	50	30	20	17	35	48
APDSHT3	45	25	30	17	35	48
APDSHT4	35	45	20	17	35	48
APDSHT5	45	40	15	17	35	48
APDSHT6	35	30	35	17	35	48

b)

	max AT (ms)	median APD_{90} (ms)	median CV (cm/s)
baseline	42.5	226.5	61.39
APDSHL1	42.5	227.25	61.38
APDSHL2	42.5	224.75	61.40
APDSHL3	42.5	225	61.46
APDSHL4	42.5	227.75	61.37
APDSHL5	42.5	226.75	61.45
APDSHL6	42.5	226	61.40
APDSHT1	42.5	226	61.44
APDSHT2	42.5	227.75	61.39
APDSHT3	42.5	226.25	61.40
APDSHT4	42.5	227	61.38
APDSHT5	42.5	227.75	61.36
APDSHT6	42.5	225.75	61.44

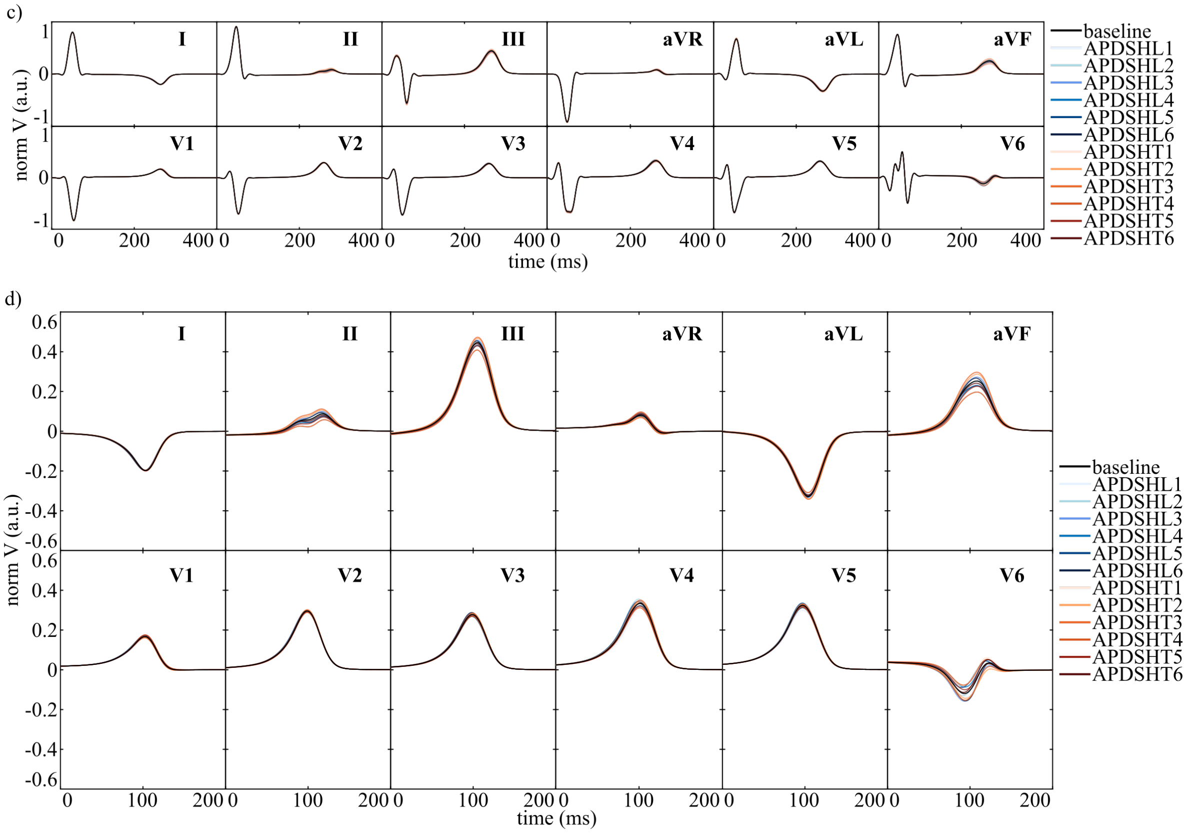

Figure 6: **Sensitivity analysis of APDSH distribution on the simulated ECG.** a- Twelve APDSH configurations were evaluated against the baseline APDSH, which was defined using transmural and longitudinal ratios adapted from non-porcine sources. Specifically, APDSH variations were generated by decreasing the size of one layer by 10% while simultaneously increasing the remaining two layers by 5% each; this was performed independently for both transmural and longitudinal directions. b- Maximum AT and median APD_{90} and CV values obtained at the anterior view of models with different APDSH configurations when simulated with a 1000 ms cycle length. c- Simulated ECGs demonstrating no variation in QRS complexes and negligible alterations in T waves across all tested APDSH ratios. d- Magnified simulated T-waves highlighting the preservation of T-wave morphology, with only minimal amplitude fluctuations primarily observed in leads II, III, aVF, and V4–V6. APDSH: Action potential duration spatial heterogeneities, ECG: Electrocardiogram, AT: Activation time, APD_{90}: Action potential duration at 90% repolarization, CV: Conduction velocity.

- *Ion-channel modulation strategy:* Regarding the choice of ion-channel modulation, our strategy was influenced by the characteristics of the specific porcine cellular model used. While prior human models have successfully used I_{Kr} and I_{Ks}, either individually or in combination, the porcine phenotype is suggested to exhibit distinct repolarization dynamics [24]. In the revised manuscript, we have

discussed this in Section 4.5, where, regarding the selection of g_{K1} to tune the APD values in the border zone, we stated:

"Following methodologies established for human models [3, 5], we reduced I_{Kr} and I_{Ks} conductances in the Gaur et al. [24] porcine model to 28.9% of their default values. This modification led to a relatively modest 21% increase in APD_{90}, whereas applying the same reduction to I_{K1} resulted in a markedly larger 63% prolongation. Given the model's greater sensitivity to the inward rectifier current, APD prolongation in this study was therefore achieved by decreasing g_{K1}."

In addition, we have added a paragraph to the Discussion of the revised manuscript regarding how the choice of g_{K1} modulation reflects both the experimental observation that APD in pigs seems to be more susceptible to I_{K1} rather than to other K^+-related ionic currents and a lack of detailed porcine-specific experimental evidence for regional channel densities. The new paragraph in the revised Section 4.3 is as follows:

"To our knowledge, this is the first computational study to reproduce experimentally observed porcine APDSH, achieved here by modifying g_{K1} in the Gaur et al. [24] model. This *in silico* mechanistic insight suggests that I_{K1} plays a more dominant role in modulating the porcine APD than other K^+-related currents typically emphasized in human models. This finding is consistent with experimental evidence showing that pigs exhibit high sensitivity to proarrhythmic effects induced by I_{K1} blockade [23], while remaining largely insensitive to I_{Kr} inhibition [25]. However, because quantitative porcine-specific data on regional ionic current densities remain limited, further experimental studies are warranted to determine whether I_{K1} modulation more accurately reflects the underlying porcine repolarization gradients."

Model Interpretation and Reporting

- Table 4 is difficult to interpret because several abbreviations and labels (e.g., s, b, a) are not defined. This prevents clear understanding of how spatial personalization was applied.

We agree with the Reviewer that Table 4 (here Table 2) was difficult to interpret due to the use of several abbreviations that were not clearly defined. To address this issue, we have added definitions of all abbreviations in the caption of Table 2, as well as in the captions of all figures and tables throughout the manuscript. In addition, we grouped the "Pig" and "Group" columns, which indicate which pigs were modeled and how, and we introduced an additional column,"Input (pig)", which explicitly reports the experimental data used in each simulation group.

Although Table 4 remains relatively complex, we consider this level of detail important to provide a clear and structured overview of the specific experimental data, modeling definitions, and simulation variables used in each simulation group. We believe that with the new additions, the table will be more clearly understood.

- The statement that "74 simulations were performed" requires clarification. Is this total across all pigs, or per animal?

Following the Reviewer's comment, we clarified that 74 simulations represent the total number of computations across all pigs. The revised phrase is now: "A total of 74 simulations across all pigs was performed using the open source software ELECTRA (v0.6.2) on the HERMES HPC cluster at the University of Zaragoza."

Similarity Metric

- The Pearson correlation coefficient is a parametric measure. If used as a statistical inference tool, normality should be tested, or a non-parametric alternative considered.

- If it is intended purely as a signal similarity score, this should be stated explicitly and not framed as a hypothesis-testing metric.

We thank the Reviewer for this comment. In this work, the Pearson correlation coefficient was not used as a statistical inference or hypothesis-testing metric, but solely as a quantitative measure of similarity between experimental and simulated signals. Therefore, no assumptions regarding normality were required. We have clarified this point in the Methods section to avoid any potential misinterpretation.

The revised phrases in Section 2.8 are: "Pearson correlation coefficients were calculated for the QRS complexes and T waves in each lead to quantify the similarity between simulated and experimental waveforms. These coefficients served to provide a normalized metric of shape similarity rather than a basis for any statistical inference."

Model Validation and Tolerances

- The manuscript would benefit from explicitly defining a use case for the model and the corresponding:

* how accuracy is assessed,
* what constitutes acceptable error for intended use, and
* whether the model meets those thresholds.

We thank the Reviewer for this suggestion, which allows us to clarify the scope and reliability of our modeling pipeline and the models we have developed.

We have expanded the Discussion by adding a new subsection titled "Model usability and preclinical implications". In this subsection, we explicitly define general use cases for the model alongside a specific application involving the assessment of arrhythmic risk in remuscularization therapy. Furthermore, we detail the methodology used to assess model accuracy and establish error tolerances for future studies. Finally, we transparently address the two specific instances where our models did not meet these predefined criteria, discussing the implications for subject-specific modeling and ways to reduce these effects. This new subsection in the revised manuscript reads:

"The developed models provide a high-fidelity *in silico* platform to investigate how healthy and MI conditions affect electrophysiological outcomes (ECG and OM) in a translational porcine model. Beyond mechanistic insights, these models may support preclinical cardiovascular applications, including the assessment of drug proarrhythmicity [24], cardiac resynchronization therapy [15], and the optimization of cardiac tissue engineering strategies. Specifically, our framework can inform the design and implantation of engineered heart tissues for remuscularization [34, 36].

Given that post-MI remuscularization is currently in the preclinical clearance phase [37, 38, 39], these models constitute valuable tools for assessing arrhythmic risk [40, 36], thereby reducing reliance on animal experimentation. To ensure suitability for this purpose, we employed a multi-modal validation strategy with predefined accuracy thresholds:

- *Depolarization and repolarization:* Accuracy was evaluated at the tissue level by comparing simulated maximum ATs, mean CVs, and mean APD_{90} values with OM data. Acceptable tolerances were defined based on experimental uncertainty and established benchmarks. For AT, we targeted a difference of ≤ 10 ms [17, 26, 41], while simulated CV and APD_{90} values were required to lie within the experimental mean $\pm$ standard deviation.

- *Activation patterns:* A qualitative agreement with experimental depolarization patterns was required, particularly during sinus rhythm where activation is not externally paced [4, 14, 17]. Benchmarks included the first epicardial breakthrough occurring in the RV area pretrabecularis and the latest activation in the posterobasal LV/RV regions [26, 41].

- *ECG morphology:* At the organ level, consistency in wave polarity, morphology, and duration was prioritized. We targeted a Pearson correlation coefficient ≥ 0.7 between experimental and simulated QRS complexes and T waves [42].

Our results show that the models successfully met the predefined thresholds whenever experimental data were available for comparison. Two specific exceptions were identified: simulated CV was higher only in the posterior view of the heart (G4 simulations), and QRS similarity was lower for pig 6 (G6 simulations), as the CS was defined using an ECG from a different subject. These discrepancies may affect the accuracy of arrhythmogenesis predictions, underscoring the need for regional CV calibration [19] and subject-specific experimental data to enable more precise digital twinning. The latter is supported by the observation that T-wave similarity nearly reached the target threshold once post-MI LV activation alterations were implemented. Overall, our cohort provides a physiologically consistent representation of the porcine phenotype under both healthy and MI conditions, offering a robust foundation for evaluating arrhythmicity through *in silico* inducibility protocols and other

translational applications."

- The authors should include a sensitivity analysis, particularly in light of the non-porcine input parameters.

In response to the Reviewer's request, we have added a sensitivity analysis to address the uncertainty associated with input parameters derived from non-porcine sources (human and canine literature). To quantify the influence of each uncertain parameter on model outputs across a biologically plausible parameter space, we performed the following simulations and obtained the corresponding results:

- *APDSH definition:* As outlined in a previous response to the Reviewer, we evaluated twelve APDSH configurations in which transmural and apicobasal ratios were varied independently. Directional variations were implemented by increasing or decreasing one region by 10%, while compensating by decreasing or increasing the remaining two regions by 5% each (see supporting Fig 6a). Variations in APDSH ratios did not affect the results at either the ECG level (QRS complex and T wave morphology) or the tissue level, including maximum AT, superficial median CV, and median APD_{90} values (see supporting Fig 6). This analysis confirms that, although some APDSH ratios were adapted from human data due to the scarcity of porcine-specific regional measurements, the primary model outputs are not critically sensitive to moderate deviations in these parameters. This observation has been added to the revised manuscript, as indicated in the previous answer to the Reviewer.
- *Ventricular and CS conductivity:* The LDC assigned to the myocardium of pig 1 was varied by $\pm$20% (supporting Fig 7). ECG-related outputs were robust, with only slightly shorter and longer QRS complex durations observed. At the tissue level, the median APD_{90} remained unchanged, while maximum AT and median CV varied by at most 4% and 10%, respectively. Similarly, the LDC assigned to the CS of pig 1 was varied by $\pm$20% (supporting Fig 8). As with myocardial LDC variations, QRS complex and T-wave polarity and morphology were preserved, with only minor changes in QRS duration. Tissue-level analysis showed variations below 10% in maximum AT, while median ventricular APD_{90} and CV values were unaffected. These results were incorporated into Section 4.2 as follows: "Despite stochastic CS variations, the preservation of QRS morphology across pigs 1-3 (same breed, weight, and age) highlights the robustness and reliability of our modeling approach. In this context, a sensitivity analysis of non-species-specific parameters demonstrated that a $\pm$20% variation in myocardial (S5 Figure) and CS (S6 Figure) conductivity resulted in, at most, a 10% change in maximum AT and CV, respectively. Notably, QRS complex morphology remained largely unaffected."
- *Post-MI I_{Na} remodeling:* g_{Na} was varied by $\pm$6% around its reduced post-MI value in the BZ of pig 6, corresponding to 32% and 44% of the default healthy value. Lower g_{Na} values produced more pronounced S-wave deflections in the QRS complex, whereas higher value attenuated the S wave. T-wave morphology and median repolarization metrics remained unchanged across g_{Na} variations. Notably, a $\pm$6% change in BZ g_{Na} induced up to 10% variation in maximum AT and median CV, highlighting the importance of calibrating this parameter using porcine-specific post-MI data when such data become available. In the revised manuscript, this outcome was incorporated to Section 4.5: "Furthermore, the characterization of I_{Na} blockade proved paramount (S7 Figure); 6% variations led to noticeable changes in S-wave morphology of simulated QRS complexes and a 10% variation in maximum BiV AT and median CV within the BZ. Due to the scarcity of porcine-specific data on post-MI ionic remodeling, excitability changes were modeled using canine patch-clamp observations of I_{Na} [32]. In contrast, structural remodeling and conductivity changes were captured using experimental DW-CMR-based anisotropy from MI pigs 7 and 14. Finally, LDC was calibrated to achieve CV agreement with OM data within the BZ."

To reflect this analysis in the manuscript, we added the figures referenced above as supporting material and expanded the "Limitations, uncertainties, and future work" section to explicitly address cross-species data usage. The revised paragraph of Section 4.8 reads as follows:

"*Cross-species data:* Although pigs are widely regarded as a robust translational model for human cardiac electrophysiology, relevant inter-species differences remain. In particular, quantitative porcine

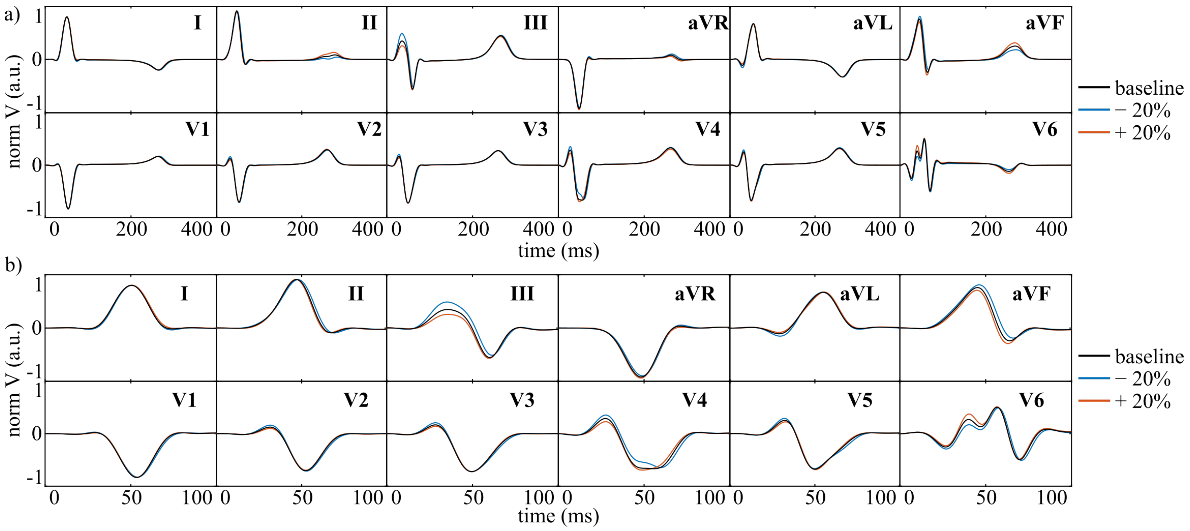

c)

	max AT (ms)	median APD_{90} (ms)	median CV (cm/s)
baseline	43	246.75	61.54
− 20%	44.75	246.75	55
+ 20%	42	246.75	67.60

Figure 7: **Sensitivity analysis of ventricular conductivity.** The baseline LDC of the healthy myocardium was varied by ±20% in pig 1. a- Complete simulated ECG signals. b- Magnified simulated QRS complexes. c- Simulated maximum AT and median APD_{90} and CV values obtained at the anterior face of the BiV model of pig 1. Simulated ECG and electrophysiological outputs were obtained under *in silico* pacing with 769 ms and 1000 ms cycle length, respectively. LDC: Longitudinal diffusion coefficient, ECG: Electrocardiogram, AT: Activation time, APD_{90}: Action potential duration at 90% repolarization, CV: Conduction velocity, BiV: Biventricular.

data of APDSH ratios, CS electrophysiology, and BZ ionic remodeling remain scarce. Consequently, several parameters were adapted from human or canine studies that reproduce qualitative electrophysiological behaviors characteristic of large mammalian hearts [28, 29, 30, 26, 31, 32, 33, 3, 5]. To assess the impact of this limitation, a sensitivity analysis was performed on APDSH ratios (S4 Figure), myocardial (S5 Figure) and CS (S6 Figure) conductivities, as well as post-MI I_{Na} remodeling (S7 Figure). At the ECG level, only minor variations were observed, including slightly shorter or longer QRS complex durations for LDC variations in the CS and myocardium, and small S-wave deflections associated with increased BZ excitability remodeling. At the tissue level, electrophysiological markers were insensitive to variations in APDSH ratios. Modifying CS and myocardial LDC by ±20% changed maximum AT and median CV by less than 10%. In contrast, ±6% variations in BZ g_{Na} resulted in changes of approximately 10% in activation-related metrics, underscoring the need for improved calibration of this parameter using porcine-specific post-MI data to further reduce output uncertainty. Overall, this sensitivity analysis suggests that most cross-species parameter choices do not compromise model robustness; however, certain parameters may introduce bias. To mitigate this effect, key outputs (AT, CV, APD_{90}, depolarization patterns, and ECG morphology) were calibrated against porcine experimental OM and ECG data, ensuring physiological consistency with the porcine phenotype."

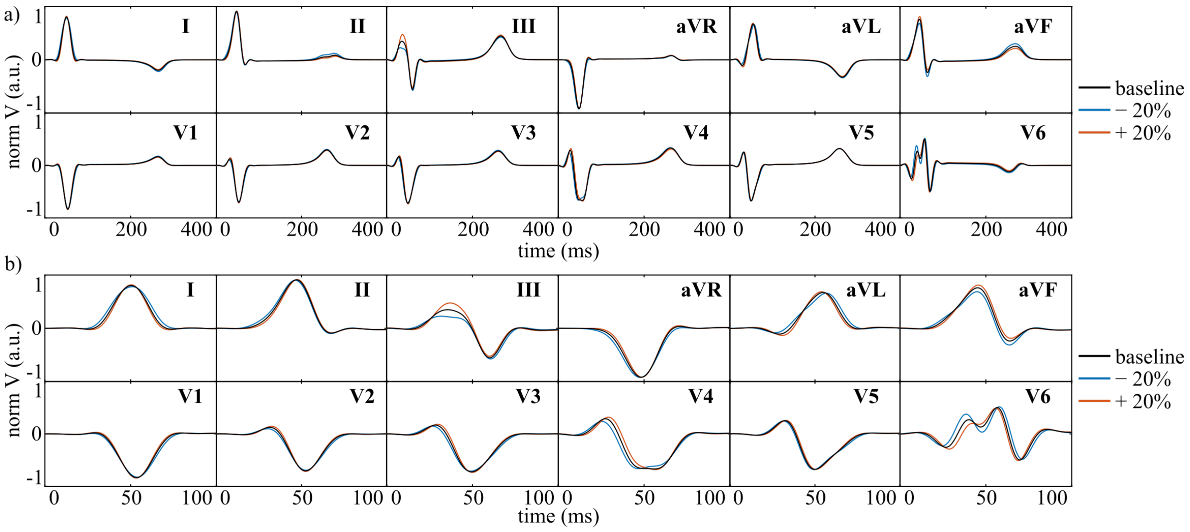

c)

	max AT (ms)	median APD$_{90}$ (ms)	median CV (cm/s)
baseline	43	246.75	61.54
− 20%	47.25	247	61.60
+ 20%	39.75	246.75	61.66

Figure 8: **Sensitivity analysis of CS conductivity.** The baseline LDC of the CS was varied by ±20% in pig 1. a- Complete simulated ECG signals. b- Magnified simulated QRS complexes. c- Simulated maximum AT and median APD$_{90}$ and CV values obtained at the anterior face of the BiV model of pig 1. Simulated ECG and electrophysiological outputs were obtained under *in silico* pacing with 769 ms and 1000 ms cycle length, respectively. CS: Conduction system, LDC: Longitudinal diffusion coefficient, ECG: Electrocardiogram, AT: Activation time, APD$_{90}$: Action potential duration at 90% repolarization, CV: Conduction velocity, BiV: Biventricular.

References

[1] Connolly AJ, Bishop MJ. Computational representations of myocardial infarct scars and implications for arrhythmogenesis. Clinical Medicine Insights: Cardiology. 2016;10s1:CMC.S39708. doi:10.4137/CMC.S39708.

[2] Mendonca Costa C, Plank G, Rinaldi CA, Niederer SA, Bishop MJ. Modeling the electrophysiological properties of the infarct border zone. Frontiers in Physiology. 2018;9:356. doi:10.3389/fphys.2018.00356.

[3] Arevalo HJ, Vadakkumpadan F, Guallar E, Jebb A, Malamas P, Wu KC, et al. Arrhythmia risk stratification of patients after myocardial infarction using personalized heart models. Nature Communications. 2016;7:11437. doi:10.1038/ncomms11437.

[4] Mendonca Costa C, Gemmell P, Elliott MK, Whitaker J, Campos FO, Strocchi M, et al. Determining anatomical and electrophysiological detail requirements for computational ventricular models of porcine myocardial infarction. Computers in Biology and Medicine. 2022;141:105061. doi:10.1016/j.compbiomed.2021.105061.

[5] Lopez-Perez A, Sebastian R, Izquierdo M, Ruiz R, Bishop M, Ferrero JM. Personalized cardiac computational models: From clinical data to simulation of infarct-related ventricular tachycardia. Frontiers in Physiology. 2019;10:580. doi:10.3389/fphys.2019.00580.

[6] Meijborg VMF, Conrath CE, Opthof T, Belterman CNW, De Bakker JMT, Coronel R. Electrocardiographic T wave and its relation with ventricular repolarization along major anatomical axes. Circulation: Arrhythmia and Electrophysiology. 2014;7:524-31. doi:10.1161/CIRCEP.113.001622.

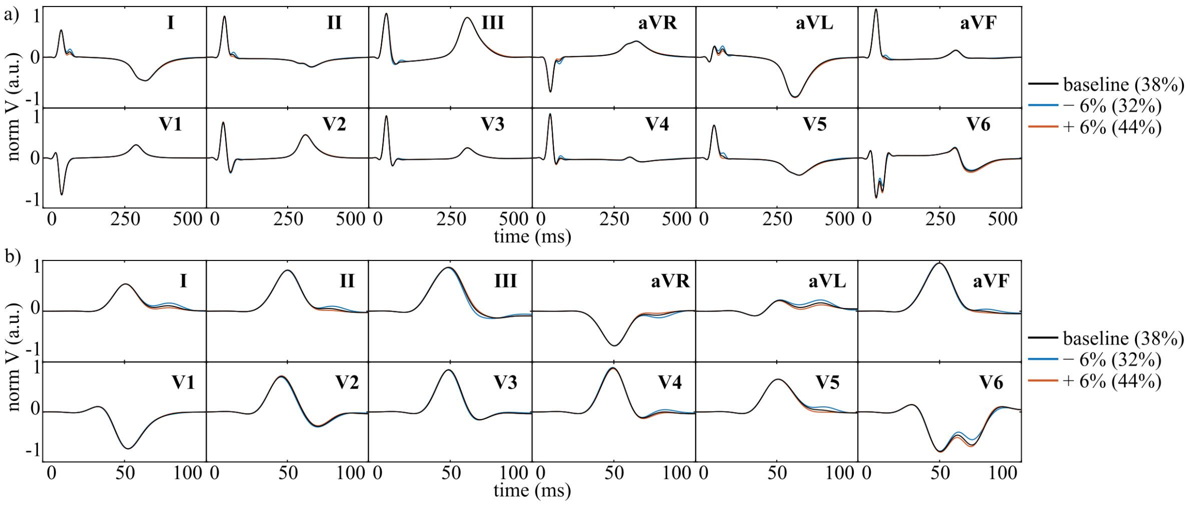

c)

	max AT (ms)	median BZ APD_{90} (ms)	median BZ CV (cm/s)
baseline (38%)	81	348.25	29.61
− 6% (32%)	89.25	345.5	27.85
+ 6% (44%)	76.5	359.5	33

Figure 9: **Sensitivity analysis of BZ I_{Na} remodeling.** The g_{Na} value applied to the BZ (38% of the healthy, default value) was varied by ±6% (32% and 44% of the healthy, default value) in pig 6. a- Complete simulated ECG signals. b- Magnified simulated QRS complexes. c- Simulated maximum ventricular AT and median APD_{90} and CV values within the BZ. Simulated ECG and electrophysiological outputs were both obtained under *in silico* pacing with 1000 ms cycle length. BZ: Border zone, I_{Na}: Sodium current, g_{Na}: Maximum conductance of the I_{Na}, ECG: Electrocardiogram, AT: Activation time, APD_{90}: Action potential duration at 90% repolarization, CV: Conduction velocity.

[7] Gillette K, Gsell MAF, Prassl AJ, Karabelas E, Reiter U, Reiter G, et al. A framework for the generation of digital twins of cardiac electrophysiology from clinical 12-leads ECGs. Medical Image Analysis. 2021;71:102080. doi:10.1016/j.media.2021.102080.

[8] Gima K, Rudy Y. Ionic current basis of electrocardiographic waveforms: A model study. Circulation Research. 2002;90:889-96. doi:10.1161/01.RES.0000016960.61087.86.

[9] Perotti LE, Krishnamoorthi S, Borgstrom NP, Ennis DB, Klug WS. Regional segmentation of ventricular models to achieve repolarization dispersion in cardiac electrophysiology modeling. International Journal for Numerical Methods in Biomedical Engineering. 2015;31:e02718. doi:10.1002/cnm.2718.

[10] Sánchez-Quintana D, Yen Ho S. Anatomía de los nodos cardíacos y del sistema de conducción específico auriculoventricular. Revista Española de Cardiología. 2003;56:1085-92. doi:10.1157/13054255.

[11] Sebastian R, Zimmerman V, Romero D, Sánchez-Quintana D, Frangi AF. Characterization and modeling of the peripheral cardiac conduction system. IEEE Transactions on Medical Imaging. 2013;32:11. doi:10.1109/TMI.2012.2221474.

[12] Pagani S, Dede' L, Manzoni A, Quarteroni A. Data integration for the numerical simulation of cardiac electrophysiology. Pacing and Clinical Electrophysiology. 2021;44:726-36. doi:10.1111/pace.14198.

[13] Rodriguez B, Carusi A, Abi-Gerges N, Ariga R, Britton O, Bub G, et al. Human-based approaches to pharmacology and cardiology: An interdisciplinary and intersectorial workshop. Europace. 2016;18:1287-98. doi:10.1093/europace/euv320.

[14] Camara O, Pop M, Rhode K, Sermesant M, Smith N, Young A, et al., editors. Statistical atlases and computational models of the heart: First international workshop, STACOM 2010, and cardiac

electrophysiological simulation challenge, CESC 2010, held in conjunction with MICCAI 2010, Beijing, China, September 20, 2010. Proceedings. vol. 6364. Springer Berlin Heidelberg; 2010. doi:10.1007/978-3-642-15835-3.

[15] Pop M, Sermesant M, Camara O, Zhuang X, Li S, Young A, et al., editors. Statistical atlases and computational models of the heart. Multi-sequence CMR segmentation, CRT-EPiggy and LV full quantification challenges: 10th international workshop, STACOM 2019, held in conjunction with MICCAI 2019, Shenzhen, China, October 13, 2019, revised selected papers. vol. 12009. Springer International Publishing; 2020. doi:10.1007/978-3-030-39074-7.

[16] Pop M, Sermesant M, Lepiller D, Truong MV, McVeigh ER, Crystal E, et al. Fusion of optical imaging and MRI for the evaluation and adjustment of macroscopic models of cardiac electrophysiology: A feasibility study. Medical Image Analysis. 2009;13:370-80. doi:10.1016/j.media.2008.07.002.

[17] Camara O, Sermesant M, Lamata P, Wang L, Pop M, Relan J, et al. Inter-model consistency and complementarity: Learning from ex-vivo imaging and electrophysiological data towards an integrated understanding of cardiac physiology. Progress in Biophysics and Molecular Biology. 2011;107:122-33. doi:10.1016/j.pbiomolbio.2011.07.007.

[18] Mincholé A, Zacur E, Ariga R, Grau V, Rodriguez B. MRI-based computational torso/biventricular multiscale models to investigate the impact of anatomical variability on the ECG QRS complex. Frontiers in Physiology. 2019;10:1103. doi:10.3389/fphys.2019.01103.

[19] Albors C, Lluch È, Gomez JF, Cedilnik N, Mountris KA, Mansi T, et al. Meshless electrophysiological modeling of cardiac resynchronization therapy—benchmark analysis with finite-element methods in experimental data. Applied Sciences. 2022;12:6438. doi:10.3390/app12136438.

[20] Vermoortele D, Olianti C, Amoni M, Giardini F, De Buck S, Nagaraju CK, et al. Precision sampling of discrete sites identified during in-vivo functional testing in the mammalian heart. Communications Engineering. 2024;3:170. doi:10.1038/s44172-024-00307-z.

[21] Kong W, Fakhari N, Sharifov OF, Ideker RE, Smith WM, Fast VG. Optical measurements of intramural action potentials in isolated porcine hearts using optrodes. Heart Rhythm. 2007;4:1430-6. doi:10.1016/j.hrthm.2007.07.002.

[22] Stankovicova T, Szilard M, De Scheerder I, Sipido KR. M cells and transmural heterogeneity of action potential configuration in myocytes from the left ventricular wall of the pig heart. Cardiovascular Research. 2000;45:952-60. doi:10.1016/S0008-6363(99)00418-6.

[23] Klein MG, Shou M, Stohlman J, Solhjoo S, Haigney M, Tidwell RR, et al. Role of suppression of the inward rectifier current in terminal action potential repolarization in the failing heart. Heart Rhythm. 2017;14:1217-23. doi:10.1016/j.hrthm.2017.04.001.

[24] Gaur N, Qi XY, Benoist D, Bernus O, Coronel R, Nattel S, et al. A computational model of pig ventricular cardiomyocyte electrophysiology and calcium handling: Translation from pig to human electrophysiology. PLoS Computational Biology. 2021;17:e1009137. doi:10.1371/journal.pcbi.1009137.

[25] Citerni C, Kirchhoff J, Olsen LH, Sattler SM, Grunnet M, Edvardsson N, et al. Inhibition of KCa2 and Kv11.1 channels in pigs with left ventricular dysfunction. Frontiers in Pharmacology. 2020;11. doi:10.3389/fphar.2020.00556.

[26] Durrer D, Van Dam RT, Freud GE, Janse MJ, Meijler FL, Arzbaecher RC. Total excitation of the isolated human heart. Circulation. 1970;41:899-912. doi:10.1161/01.CIR.41.6.899.

[27] Schmidt A, Azevedo CF, Cheng A, Gupta SN, Bluemke DA, Foo TK, et al. Infarct tissue heterogeneity by magnetic resonance imaging identifies enhanced cardiac arrhythmia susceptibility in patients with left ventricular dysfunction. Circulation. 2007;115:2006-14. doi:10.1161/CIRCULATIONAHA.106.653568.

[28] Drouin E, Charpentier F, Gauthier C, Laurent K, Le Marec H. Electrophysiologic characteristics of cells spanning the left ventricular wall of human heart: Evidence for presence of M cells. Journal of the American College of Cardiology. 1995;26:185-92. doi:10.1016/0735-1097(95)00167-X.

[29] Pueyo E, Corrias A, Virág L, Jost N, Szél T, Varró A, et al. A multiscale investigation of repolarization variability and its role in cardiac arrhythmogenesis. Biophysical Journal. 2011;101:2892-902. doi:10.1016/j.bpj.2011.09.060.

[30] Stewart P, Aslanidi OV, Noble D, Noble PJ, Boyett MR, Zhang H. Mathematical models of the electrical action potential of Purkinje fibre cells. Philosophical Transactions of the Royal Society A: Mathematical, Physical and Engineering Sciences. 2009;367:2225-55. doi:10.1098/rsta.2008.0283.

[31] Spach MS, Miller WT, Geselowitz DB, Barr RC, Kootsey JM, Johnson EA. The discontinuous nature of propagation in normal canine cardiac muscle. Evidence for recurrent discontinuities of intracellular resistance that affect the membrane currents. Circulation Research. 1981;48:39-54. doi:10.1161/01.RES.48.1.39.

[32] Pu J, Boyden PA. Alterations of Na^+ currents in myocytes from epicardial border zone of the infarcted heart. Circulation Research. 1997;81:110-9. doi:10.1161/01.RES.81.1.110.

[33] Potse M, Dubé B, Richer J, Vinet A, Gulrajani RM. A comparison of monodomain and bidomain reaction-diffusion models for action potential propagation in the human heart. IEEE Transactions on Biomedical Engineering. 2006;53:11. doi:10.1109/TBME.2006.880875.

[34] Rosales RM, Mountris KA, Oliván-Viguera A, Pérez-Zabalza M, Cedillo-Servin G, Iglesias-García O, et al. Experimentally-guided *in silico* design of engineered heart tissues to improve cardiac electrical function after myocardial infarction. Computers in Biology and Medicine. 2024;171:108044. doi:10.1016/j.compbiomed.2024.108044.

[35] Zaniboni M, Pollard AE, Yang L, Spitzer KW. Beat-to-beat repolarization variability in ventricular myocytes and its suppression by electrical coupling. American Journal of Physiology-Heart and Circulatory Physiology. 2000;278:H677-87. doi:10.1152/ajpheart.2000.278.3.H677.

[36] Rosales RM, Ríos-Muñoz GR, Sánchez de la Nava AM, Fernández-Santos ME, Bermejo J, Doblaré M, et al. In silico assessment of arrhythmic risk following the implantation of engineered heart tissues in porcine hearts with varying infarct locations [Preprint]. bioRxiv; 2025 [cited 2026 January 01]. Available from: `https://doi.org/10.1101/2025.11.13.688201`.

[37] Jebran AF, Seidler T, Tiburcy M, Daskalaki M, Kutschka I, Fujita B, et al. Engineered heart muscle allografts for heart repair in primates and humans. Nature. 2025;639:503-11. doi:10.1038/s41586-024-08463-0.

[38] Marchiano S, Nakamura K, Reinecke H, Neidig L, Lai M, Kadota S, et al. Gene editing to prevent ventricular arrhythmias associated with cardiomyocyte cell therapy. Cell Stem Cell. 2023;30:396-414.e9. doi:10.1016/j.stem.2023.04.010.

[39] Romagnuolo R, Masoudpour H, Porta-Sánchez A, Qiang B, Barry J, Laskary A, et al. Human embryonic stem cell-derived cardiomyocytes regenerate the infarcted pig heart but induce ventricular tachyarrhythmias. Stem Cell Reports. 2019;12:967-81. doi:10.1016/j.stemcr.2019.04.005.

[40] Yu JK, Liang JA, Franceschi WH, Huang Q, Pashakhanloo F, Sung E, et al. Assessment of arrhythmia mechanism and burden of the infarcted ventricles following remuscularization with pluripotent stem cell-derived cardiomyocyte patches using patient-derived models. Cardiovascular Research. 2022;118:1247-61. doi:10.1093/cvr/cvab140.

[41] Wyndham CR, Meeran MK, Smith T, Saxena A, Engelman RM, Levitsky S, et al. Epicardial activation of the intact human heart without conduction defect. Circulation. 1979;59:161-8. doi:10.1161/01.CIR.59.1.161.

[42] Camps J, Berg LA, Wang ZJ, Sebastian R, Riebel LL, Doste R, et al. Digital twinning of the human ventricular activation sequence to clinical 12-lead ECGs and magnetic resonance imaging using realistic Purkinje networks for *in silico* clinical trials. Medical Image Analysis. 2024;94:103108. doi:10.1016/j.media.2024.103108.
