# Supplementary material for: Integrated multi-modal data analysis for computational modeling of healthy and location-dependent myocardial infarction conditions in porcine hearts
Source: PLoS Comput Biol. 2026 Mar 16;22(3):e1013688. doi: 10.1371/journal.pcbi.1013688 (PMC13008256; doi:10.1371/journal.pcbi.1013688)
Supplement: Attachment — Submitted filename: Response_to_Reviewers_auresp_2.pdf [file pcbi.1013688.s009.pdf]

February 13, 2026

Re: Re-submission of manuscript "Integrated multi-modal data analysis for computational modeling of healthy and location-dependent myocardial infarction conditions in porcine hearts", with reference PCOMPBIOL-D-25-02241

Ricardo M. Rosales
University of Zaragoza
Zaragoza, Spain
rrosales@unizar.es

Editor in Chief
PLoS Computational Biology

Dear Editor,

We sincerely thank you for the opportunity to revise our manuscript entitled "Integrated multi-modal data analysis for computational modeling of healthy and location-dependent myocardial infarction conditions in porcine hearts". We greatly appreciate the careful evaluation and constructive feedback provided by the Reviewers. We believe the manuscript has been significantly strengthened by incorporating the analysis of beat-to-beat variability and its implications for our modeling framework, as well as by more clearly articulating the biological questions our work intends to address.

Below, we provide a detailed point-by-point response (in blue) to the Reviewers' comments, clearly indicating the corresponding modifications. In the "Revised Manuscript with Track Changes", revisions from the first round are shown in red, while changes introduced in this second revision are highlighted in blue for clarity.

This revised version has been prepared in consultation with all co-authors, and each has approved the final manuscript. We thank you and the Reviewers once again for your time, consideration, and valuable feedback.

Yours sincerely,
Ricardo M. Rosales, on behalf of all authors

Revision of manuscript PCOMPBIOL-D-25-02241

Editor comments:

Please confirm the materials are available in zenodo, reinforce the biological understanding that is gleaned and can be obtained using this computational advance (that was not before possible), and attend to the remaining concern of Reviewer 1.

We thank the Editor for this feedback. We confirm that the datasets supporting the findings of this study are openly available in Zenodo repositories. The specific datasets can be openly accessed via the links provided in the "Data availability" section and are listed below:

- Experimental dataset: `https://doi.org/10.5281/zenodo.18223187`
- Simulation dataset: `https://doi.org/10.5281/zenodo.17415592`

In response to the remaining concerns of Reviewer 1 (beat-to-beat variability) and 2 (biological understanding/questions), we provide detailed answers in the following.

Response to Reviewer's comments

Reviewer 1:

Thank your for addressing my points and improving the manuscript.

We are grateful to the Reviewer for the revisions and for the helpful and constructive comments, which we addressed as follows.

I have one remaining point concerning beat-to-beat variability (#1 in my initial review):
I think it would be important to mention the experimental beat-to-beat variability that remains after quite strict beat selection (median standard deviation across pixels: 20.5ms for activation and 51.5ms for APD90 in pig 13) to i) put the remaining deviation during parameter tuning into perspective (doesn't make sense to aim for sub-milisecond precision during tuning when measurement variability/uncertainty is in the range of dozens of miliseconds); ii) highlight that while some of the observed variability is probably due to measurement uncertainty, there is very likely a contribution of intrinsic beat-to-beat variability as well, which is not considered in the model.

We thank the Reviewer for this insightful suggestion. We agree that highlighting the observed beat-to-beat variability is essential to provide the necessary context for interpreting the simulation results, particularly regarding the precision of parameter tuning and the deterministic nature of the model.

In the revised Section 2.3, we have now added a quantitative description of the experimental beat-to-beat variability to better characterize the uncertainty inherent in the OM data:

"Median AT and APD_{90} values across beats were used to generate AT and APD_{90} maps. Despite selecting consecutive beats with identical beating rates to minimize beat-to-beat variability in depolarization and repolarization measurements, residual variability remained. This was particularly evident in repolarization times near the infarct and in regions with significant fat coverage, such as the interventricular septum. For example, in pig 13 ($n = 11$ beats, sinus rhythm), the median standard deviation across pixels was 20.5 and 51.5 ms for ATs and APD_{90} values, respectively, whereas in pig 10 ($n = 4$ beats, sinus rhythm), the corresponding values were 4.8 and 4.6 ms for ATs and APD_{90} values, respectively. Given this variability, median values were considered a more robust and representative summary of the OM data. This level of experimental uncertainty, which can reach several tens of milliseconds, defines a realistic limit for parameter tuning."

Furthermore, in the "Limitations, uncertainties, and future work" section, we have addressed the fact that the intrinsic variability was not accounted for in the simulations. This addition clarifies that our model-experiment agreement is based on median trends rather than individual beat captures:

"Our simulations demonstrated strong agreement with the median OM findings rather than on a per-beat basis, as the experimental results exhibited inherent beat-to-beat variability that was not

captured by our deterministic modeling framework. Future studies could incorporate stochastic modeling to better reproduce the variability observed in the experimental data, which was particularly prominent in specific regions and samples (see Section 2.3)."

Reviewer 2:

My concerns have largely been addressed.

We are grateful to the Reviewer for the revisions and for the helpful and constructive comments, which we have addressed as follows.

The manuscript would be further strengthened by more clearly articulating the specific biological question(s) that this modeling framework is intended to address.

We agree with the Reviewer and have revised the manuscript accordingly. We now explicitly articulate the biological questions and the mechanistic understanding motivating this work. These clarifications emphasize that the proposed framework is not merely a technical pipeline, but a tool designed to elucidate why depolarization and repolarization unfold as they do in the porcine heart, in both healthy and MI conditions, and across tissue- and organ-level scales. The revised paragraph at the end of the Introduction now reads:

"In this context, this work has four main objectives: 1) to define a reproducible pipeline for the development and calibration of biventricular (BiV) electrophysiological models in healthy and MI pig hearts by processing cardiac signals and images from *in vivo* and *ex vivo* pig experiments; 2) to identify the critical parameters required to *in silico* reproduce both species-specific (porcine) and subject-specific (individual pig) electrical characteristics in healthy and MI conditions; 3) to provide openly available, state-of-the-art BiV porcine models for use in preclinical cardiovascular studies (e.g., drug cardiotoxicity, remuscularization assessment), thereby reducing reliance on animal experimentation; 4) to demonstrate the potential of these experimentally guided *in silico* models for mechanistic analysis of infarction-induced electrocardiogram (ECG) alterations across multiple infarct locations.

Beyond these technical objectives, the framework is designed to address key biological questions in both healthy and MI conditions. In the healthy state, we investigate: (i) how CS architecture and depth shape ventricular activation, and the functional implications of the deeper CS observed in pigs; (ii) the extent to which regional repolarization differences contribute to T-wave formation, assessing the presence of relatively limited yet existing repolarization heterogeneity in the porcine heart; and (iii) how fiber orientation shapes depolarization and repolarization patterns at both tissue and organ scales.

In the MI state, we shed light on: (i) whether post-MI alterations in ionic currents, particularly the inward rectifier potassium current and fast sodium current, and reduced cell-to-cell coupling constitute the primary mechanisms driving the post-MI electrophysiological phenotype; and (ii) how MI-induced changes in activation translate into ECG alterations. By linking these infarct-induced modifications to observed ECG changes across different infarct locations and extents, we provide mechanistic insight into ECG variability that is difficult to obtain experimentally in large-animal models."
